# Mutations in *NEK1* cause ciliary dysfunction as a novel pathogenic mechanism in amyotrophic lateral sclerosis

**DOI:** 10.1186/s13024-025-00848-7

**Published:** 2025-05-20

**Authors:** Min-Young Noh, Seong-il Oh, Young-Eun Kim, Sun Joo Cha, Wonjae Sung, Ki-Wook Oh, Yurim Park, Ji Young Mun, Chang-Seok Ki, Minyeop Nahm, Seung Hyun Kim

**Affiliations:** 1 Department of Neurology, College of Medicine, Hanyang University, 222, Wangsimni-ro, Seongdong-gu, Seoul, 04763 Republic of Korea; 2 Department of Neurology, Kyung Hee University Medical Center, Seoul, Republic of Korea; 3 Department of Laboratory Medicine, College of Medicine, Hanyang University, Seoul, Republic of Korea; 4 Dementia Research Group, Korea Brain Research Institute, Daegu, Republic of Korea; 5 Neural Circuit Research Group, Korea Brain Research Institute, Daegu, Republic of Korea; 6 Department of Biomedical Science, School of Medicine, Kyungpook National University, Daegu, Republic of Korea; 7 Green Cross Genome Corporation, Yongin, Republic of Korea; 8 Cell Therapy Center, Hanyang University Hospital, Seoul, Republic of Korea

**Keywords:** Amyotrophic lateral sclerosis, *NEK1*, Primary cilia, Cell cycle, Microtubule, Mitochondria, DNA damage response

## Abstract

**Background:**

Neuronal primary cilia, vital for signaling and cell-cycle regulation, have been implicated in maintaining neuronal identity. While a link between primary ciliary defects and neurodegenerative diseases is emerging, the precise pathological mechanisms remain unclear.

**Methods:**

We studied the genetic contribution of *NEK1* to ALS pathogenesis by analyzing the exome sequences of 920 Korean patients with ALS. To understand the disease contribution of *NEK1* variants in ALS, we performed a series of functional studies using patient fibroblasts focusing on primary cilia and microtubule-related phenotypes. In addition, these findings were validated in iPSC-derived motor neurons (iPSC-MNs).

**Results:**

*NIMA-related kinase 1* (*NEK1*), a gene encoding a serine/threonine kinase involved in cell cycle regulation, has been identified as a risk gene for amyotrophic lateral sclerosis (ALS). Here, we report that mutations in *NEK1* cause primary ciliary abnormality, cell cycle re-entry, and disrupted tubulin acetylation in ALS. We analyzed the whole-exome sequences of 920 Korean patients with sporadic ALS and identified 16 *NEK1* variants in 23 patients. We found that two novel variants, p.E853Rfs*9 and p.M1?, reduced NEK1 expression, resulting in loss-of-function (LOF) and one synonymous splicing variant (p.Q132=) exhibited an aberrant isoform lacking exon 5. All three *NEK1* variants exhibited abnormal primary ciliary structure, impaired sonic hedgehog signaling, and altered cell-cycle progression. Furthermore, the ALS-linked variants induced intracellular calcium overload followed by Aurora kinase A (AurA)-histone deacetylase (HDAC)6 activation, resulting in ciliary disassembly. These defects were restored by treatment with the intracellular Ca^2+^ chelator, BAPTA. We also found that *NEK1* variants cause decreased α-tubulin acetylation, mitochondrial alteration, and impaired DNA damage response (DDR). Notably, drug treatment to inhibit HDAC6 restored the NEK1-dependent deficits in patient fibroblasts. And, we confirmed that data found in patient fibroblasts were reproduced in iPSC-MNs model.

**Conclusions:**

Our results suggest that NEK1 contributes to ALS pathogenesis through the LOF mechanism, and HDAC6 inhibition provides an attractive therapeutic strategy for *NEK1* variants associated ALS treatment.

**Supplementary Information:**

The online version contains supplementary material available at 10.1186/s13024-025-00848-7.

## Background

Amyotrophic lateral sclerosis (ALS) is characterized by the selective degeneration of motor neurons (MNs) in the brain and spinal cord [1]. Genes associated with ALS are involved in various biological processes, including proteostasis, RNA metabolism, cytoskeletal dynamics, nucleocytoplasmic transport, and neuroinflammation [1, 2]. Despite extensive research proposing these cellular pathways’ role in MNs degeneration, ALS pathogenesis remains unclear. Recent genetic insights, particularly through whole exome sequencing (WES), have identified *NIMA-related kinase 1* (*NEK1)* (a multifunctional kinase involved in the cell cycle, the DNA damage response (DDR), and primary ciliogenesis) as an ALS risk gene in multiple ethnic groups [3–14]. NEK1’s functional and biochemical interaction with another ALS risk gene, *C21orf2* [3, 7, 15] which plays a role in regulating primary cilia and DDR [16, 17] suggests the potential importance of ciliary dysfunction as a novel disease mechanism in ALS.

Primary cilia are microtubule-based, non-motile organelles found in most mammalian cell types, including neurons and astrocytes [18]. They coordinate a series of signaling pathways, such as G protein-coupled receptor-mediated sonic hedgehog (Shh) signaling. These cilia are associated with cell cycle progression in dividing cells, typically assembling during the G0/G1 phase and disassembling before mitosis [19–21]. Additionally, a link between primary cilia and DDR, autophagy, and mitochondria has been recently indicated [22–24]. Primary cilia in postmitotic and terminally differentiated neurons are relatively stable and do not undergo de novo ciliogenesis. Given the essential role of the primary cilia, neuronal ciliary homeostasis should be tightly regulated to maintain neuronal identity and health. Supporting this idea, documented evidence indicates that the shortening and loss of primary cilia can trigger aberrant cell cycle re-entry, resulting in neuronal cell death. Conversely, maintaining the stability of neuronal primary cilia has a neuroprotective effect [25]. Mutations involving the structural or functional abnormalities of the primary cilia cause genetic multisystemic human disorders known as ciliopathies [26]. Additionally, the alterations in ciliary morphology and signaling are associated with aging and pathological conditions, such as Alzheimer’s disease, Parkinson’s disease (PD), and ALS [27, 28]. Some studies on ALS have revealed that the primary cilia of spinal MNs are reduced in the ALS animal models expressing mutant SOD1, and activation of Shh signaling is cytoprotective in the ALS cellular model [29, 30]. Intriguingly, expression studies using postmortem tissues from patients with ALS have demonstrated increased immunoreactivity of G1 to S phase cell cycle regulators, although whether this is directly related to the primary cilia defects is unknown [31].

*NEK1* mutations are linked to developmental ciliopathy and ALS. In humans, homozygous *NEK1* loss-of-function (LOF) mutations lead to autosomal recessive lethal osteochondrodysplasia, a severe early developmental disorder known as short-rib polydactyly syndrome type II [32, 33]. Contrastingly, most ALS-associated *NEK1* mutations are heterozygous and include LOF variants such as frameshift, stop-gain, and splicing variants, as well as missense variants [5, 6, 34, 35]. *NEK1* mutations in ALS are manifested through impaired DNA damage repair in iPSC-derived motor neurons (iPSC-MNs) from patients [36]. Furthermore, the LOF of *NEK1* was recently found to disrupt microtubule homeostasis and nucleocytoplasmic transport [37]. However, whether ciliary dysfunction caused by *NEK1* mutations contributes to ALS pathogenesis remains unclear. We enrolled patients with *NEK1* LOF variants in a cohort of Korean ALS patients and conducted functional studies focusing on primary cilia- and microtubule-related phenotypes to investigate the pathogenic potential of *NEK1* variants in ALS. Our findings demonstrated that ALS-linked *NEK1* variants induced calcium-dependent activation of the Aurora kinase A (AurA)-histone deacetylase (HDAC) 6 pathway, leading to compromised primary cilia and impaired tubulin acetylation. Additionally, treatment with an HDAC6 inhibitor restored primary cilia- and microtubule-associated defects in *NEK1* variant-carrying patient fibroblasts. Moreover, those findings were reproduced in iPSC-MNs. Thus, primary ciliary dysfunction could be explored as a novel disease mechanism in ALS pathology, presenting a potential target for therapeutic interventions.

## Methods

### Clinical data of the participants

We reviewed the genetic and clinical data of 23 patients with ALS who carried the *NEK1* variants between January 2015 and December 2020 from the ALS cohort and biobank of the Hanyang University Hospital in Seoul, Korea. Only participants carrying *NEK1* variants expected to have LOF and whose skin fibroblasts were available in the biobank were enrolled in this study. ALS diagnosis was based on the revised El Escorial criteria [38]. Patients with clinically definite, probable, probable laboratory-supported, or possible ALS were recruited. During this period, most cases were sporadic; therefore, we decided to include only sporadic ALS (sALS) cases in this study. Therefore, 920 Korean patients with sALS were enrolled. Clinical measures included age, sex, ALS family history, symptom onset region, initial ALS functional rating scale revised (ALSFRS-R) score, and calculated progression rate (delta-FS [(48-ALSFRS-R score at visit)/duration from onset to visit (months)]) [39]. We collected clinical information and family histories by interviewing participants and their caregivers. Additionally, the biobank skin fibroblasts from healthy controls were used to acquire wild-type data. Written informed consent was obtained from all participants or their legal representatives. The Institutional Review Board of Hanyang University Hospital (#HYUH 2011-08-010, HYUH 2017-01-043-002) approved the study protocol.

### Genetic analyses

Genomic DNA was extracted from peripheral blood leukocytes using the Wizard Genomic DNA Purification Kit, according to the manufacturer’s instructions (Promega, Madison, WI, USA). Variants of *NEK1* and other ALS-related genes were screened using next-generation sequencing. A TruSight^TM^ One Sequencing Panel (Illumina Inc., San Diego, CA) or an Agilent SureSelect all Exon kit 50Mb (Agilent, Santa Clara, CA) were used to prepare sequencing libraries according to the manufacturer’s instructions. The flow cell was loaded onto either a MiSeq or NextSeq 500 sequencing system (Illumina) for sequencing with 2× 100 bp read lengths. The reads were mapped to the GRCh37/hg19 build using the Burrows–Wheeler Aligner and the variants were called using GATK. All variants with allele frequencies > 0.001 were filtered out, based on various public databases, including the genome aggregation database (gnomAD, https://gnomad.broadinstitute.org, ver.4.1.1). The mean read depth of *NEK1* exceeded 115.8×, and over 98.5% of the *NEK1* coding sequence was covered by at least 10 independent sequence reads across all samples. For all identified variants in the *NEK1* gene using primers designed by the authors, Sanger sequencing validation was performed. All identified variants were classified according to the guidelines of the American College of Medical Genetics and Genomics and Association for Molecular Pathology (ACMG/AMP) [40] and recommendations by ClinGen (https://clinicalgenome.org/working-groups/sequence-variant-interpretation/).

### Generating patient-derived fibroblasts and cell culture

Adult human fibroblasts were extracted from forearm skin using a punch biopsy. The fibroblasts were cultured at 37℃ with 5% CO_2_ in media containing Dulbecco’s modified Eagles medium (DMEM), non-essential amino acids (Gibco, Carlsbad, CA, USA), sodium bicarbonate (Sigma-Aldrich), and 1% (vol/vol) penicillin/streptomycin/fungizone (Cellgro), supplemented with 20% fetal bovine serum (FBS). In all experiments, the cells were passage-matched (<10 passages). SH-SY5Y and NSC-34 cells were grown in DMEM supplemented with 10% heat-inactivated FBS and antibiotics. The cells were cultured under serum starvation for 48 h for primary cilia formation. To induce DNA damage, cells were treated with 20 μM etoposide (Sigma) for 1 h to the growth medium or exposed to 20 J/m^2^ ultraviolet (UV) irradiation (UVC, 254 nm) in a HL2000 Hybrilinker chamber (UVX radiometer, UVP) after removing the medium. Subsequently, the cells were promptly overlaid with fresh warm medium and returned to a 37 °C incubator for 24 h. For the molecular biology study, the entire *NEK1* open reading frame (ORF) was PCR-amplified from the total human cDNAs and cloned into the pEGFP-C1 vector (Clontech) to generate the pEGFP-NEK1-WT. All *NEK1* variants were generated by site-directed mutagenesis using the EZ change site-directed mutagenesis kit (Enzynomics) according to the manufacturer’s instructions. The cells were transfected using FuGENE HD transfection reagent (Promega) according to the manufacturer’s instructions for the transient expression of the *NEK1* constructs. The cells were treated with the smoothened agonist SAG (Calbiochem, Darmstadt, Germany) for 24 h to activate the sonic hedgehog (Shh) signaling pathway. For *NEK1* silencing, predesigned human *NEK1* small interfering RNA (siRNA) and control siRNA (Santa Cruz Biotechnology, Dallas, TX; sc-106907) were transfected into fibroblasts or SH-SY5Y cells using RNAiMAX (Life Technologies) according to the manufacturer’s instructions. Human fibroblasts were treated with 1 μM tubastatin A (Sigma-Aldrich; St. Louis, MO, USA) for 24 h at 37 °C for HDAC6 inhibitor treatment. For *NEK1* overexpression, fibroblasts were transduced with Human NEK1 (NM_012224) Tagged ORF Clone Lentiviral Particle or Lenti ORF control particles of pLenti-C-mGFP (OriGene Technologies, Rockville, MD, USA), according to the manufacturer’s protocol.

### Differentiation of iPSCs to MNs

Control iPSC line, KOLF2.1 J (JIPSC1000) were acquired from The Jackson Laboratory. iPSCs were grown in StemFlex™ medium consisting of StemFlex™ Basal Medium (Gibco) and StemFlex™ supplement (Gibco) in 6-well plates coated with Matrigel (Corning) for 3–5 days. The iPSCs were detached using Accutase solution (Innovative Cell Technology). Differentiation of iPSCs into MNs was performed as previously described [41, 42]. Briefly, embryoid bodies (EBs) were formed in an ultra-low attachment (ULA) 6-well plates (Corning). On day 0, the medium was changed to EBs medium containing advanced DMEM-F12 (Gibco)/neurobasal (Gibco) 50:50 medium supplemented with 1X N2 (Gibco), 1X B27 (Gibco), 1X GlutaMax (Gibco), 100 μM 2-mercaptoethanol (Gibco), 1X penicillin-streptomycin (Gibco), 10 ng/ml bFGF (Gibco), 10 μM Y-27632 dihydrochloride (TOCRIS), 10 μM SB431542 (TOCRIS), 3 μM CHIR99021 (TOCRIS), 100 nM LDN193189 (Sigma-Aldrich) and 30 μg/ml ascorbic Acid (Sigma-Aldrich). From day 2 to day 6, 100 nM retinoic acid (Sigma-Aldrich), 500 nM Purmorphamine (Sigma-Aldrich) and 500 nM smoothened agonist (SAG) (Merck) were additionally added to the medium. Y-27632 dihydrochloride and bFGF were then removed from the medium. On day 7, 10 ng/ml BDNF (Peprotech) was added to the medium. SB431542, CHIR99021, and LDN193189 were then removed from the medium. From day 9 to day 13, 10 μM DAPT (TOCRIS) was additionally added to the medium. On day 14, 10 ng/ml GDNF (Gibco) was added to the medium. DAPT was then removed from the medium. Half of the medium was changed every 2–3 days. On day 16, the EBs were dissociated enzymatically into single cells with 0.05% trypsin (Gibco) and straining them with a 40 μm cell strainer (pluriSelect). Then cells were counted and seeded onto 96-well imaging plate (5x10^4^ cells/well, Cellvis) or 12-well plate (8x10^5^ cells/well) coated with Laminin/Poly-Ornithine (Sigma-Aldrich). MN medium consisted of neurobasal, non-essential amino acids (Gibco), GlutaMAX, 100 μM 2-mercaptoethanol, penicillin-streptomycin. MN medium was supplemented with 10 ng/ml GDNF, 10 ng/ml BDNF, 10 ng/ml CNTF (Invitrogen), 10 ng/ml IGF-1 (R&D Systems), 100 nM retinoic acid, 30 μg/ml ascorbic acid, N2, and B27. The following day, cells were cultured in MN medium supplemented with 5 µM aphidicolin (Cell Signaling). After 24 h, medium was replaced with fresh media without aphidicolin. MNs were allowed to differentiate until analysis. MNs were fixed for immunofluorescence after 30 days of differentiation. Knockdown of *NEK1* was performed in iPSC-MNs using Accell SMARTPool siRNA against *NEK1* (Horizon Discovery Cat. E-004864-00-0050) and non-targeting Control Pool (Horizon Discovery Cat. D-001910-10-50) for control. Treatment was performed on day 25 at a concentration of 1 µM for 5 days.

### Cell fraction

The mitochondrial fraction was isolated from fibroblasts using a Mitochondria/Cytosol Fractionation Kit according to the manufacturer’s instructions (BioVision, Milpitas, CA, USA). Briefly, the fibroblasts were gently rinsed with ice-cold tris-buffered saline (TBS). After collecting the cells by centrifugation at 600 × *g* for 5 min at 4 °C, the cells were resuspended in a cytosol extraction buffer mixture containing DTT and protease inhibitors (Sigma-Aldrich) and incubated on ice for 10 min. Cells were homogenized in an ice-cold tissue grinder. Mitochondrial and cytosolic fractions were segregated after centrifugation at 10,000 × *g* for 30 min at 4 °C.

### Western blotting

Cells were washed twice with PBS and incubated for 10 min on ice in radioimmunoprecipitation assay (RIPA) buffer with proteinase and phosphatase inhibitors as the RIPA-soluble fractions. Protein concentrations were determined using the bicinchoninic acid assay and standardized. Equal protein amounts were analyzed by western blotting using the indicated antibodies. Primary antibodies included anti-NEK1 (1:1000, sc-398813, Santa Cruz Biotechnology), anti-GFP (1:1000, A11122, Thermo Scientific), anti-CDK4 (1:1000, sc-23896, Santa Cruz Biotechnology), anti-Cyclin D1 (1:1000, sc-20044, Santa Cruz Biotechnology), anti-RB (1:1000, GTX100545, GeneTex), anti-p-RB (S249+T252) (1:1000, sc-377528, Santa Cruz Biotechnology), anti-phospho-Aurora A (T288) (1:1000, MA5-14904, Invitrogen), anti-Aurora A (1:1000, ab13824, Abcam), anti-acetylated α-tubulin (1:1000, T6793, Sigma-Aldrich), anti-α-tubulin (1:1000, 9026, Sigma-Aldrich), anti-cytochrome c (1:1000, ab110325, Abcam), anti-caspase 3 (1:500, 9662s, Cell Signaling), anti-phospho-γH2AX (S139) (1:1000, ab26350, Abcam), anti-H2AX (1:1000, 2595, Cell Signaling Technology), anti-Chk1 (1:1000, sc-8408, Santa Cruz Biotechnology), anti-phospho-Chk1 (S345) (1:1000, 2348s, Cell Signaling Technology), anti-VDAC1 (1:1000, sc-390996, Santa Cruz Biotechnology), Anti-VDAC1/2/3 (1:1000, ab15895, Abcam), and anti-GAPDH antibody (1:1000, Santa Cruz Biotechnology). Membranes were subsequently probed with horseradish peroxidase-conjugated secondary antibodies (Santa Cruz Biotechnology). A West-Q Chemiluminescent Substrate Plus Kit (GenDEPOT, Barker, TX, USA) was used for visualizing the immunoreactive bands. The same membranes were re-probed with GAPDH as an internal control.

### Quantitative polymerase chain reaction (qPCR) and reverse transcription (RT)-PCR analysis

Gene expression in cells was measured using qPCR as previously described [43]. Briefly, total RNA was extracted using TRIzol Reagent (Invitrogen) and reverse-transcribed using a High-Capacity cDNA Reverse Transcription Kit (Applied Biosystems). qPCR analysis was performed using the SYBR Green PCR Master Mix (Applied Biosystems) with primers, and the data were normalized to *GAPDH* expression levels. The following primers were used: *NEK1* (Qiagen, Germany, PPH19690A), *GLI* (PPH00153A), *CDK4* (PPH00118F), *Cyclin D1* (*CCND1*; PPH00128F), *E2F1* (PPH00136G), *IFT80* (PPH13921A), *IFT20* (PPH07246A), *DYNC2H1* (PPH08755A),* BBS1* (PPH11950A), *BBS2* (PPH17110A), *ARL6* (PPH14191B), *OFD1* (PPH07414A), *OCRL* (PPH11757B), *TUBA1A* (F-GACGACTCCTTCACCACCTTC, R-GCATAGTTGTTGGCAGCATCC),* TUBB* (PPH17836A), and *GAPDH* (PPH00150F).

For splicing analysis of the c.396G>A (p.Q132=) *NEK1* variant, total RNA was extracted from fibroblasts using TRIzol, reverse transcribed into cDNA with random hexamers using the SuperScript III First Strand Synthesis Kit (Invitrogen), and analyzed by RT-PCR. Semi-quantitative analysis of *NEK1* splicing isoforms was performed using primers targeting exons 4–6. Primers specific to exons 4 (5′-AAAATGGCTCTCTCTACATA-3′) and 6 (5′-GATATTCTTGAGATCGT-3′) were used in the RT-PCR analysis and the amplicons were visualized in a 2% agarose gel and the intensity of the bands quantified with ImageJ software.

### Immunocytochemistry

Cultured cells were fixed with 4% formaldehyde in PBS for 20 min at room temperature (RT), permeabilized with 0.2% Triton X-100 in PBS for 15 min, and blocked with 1% bovine serum albumin (BSA) in PBS for one hour. Cells were then incubated with primary antibodies at 4 ℃ overnight and were labeled with secondary antibodies for 60 minutes at RT, followed by counterstaining with 4’,6-diamidino-2-phenylindole (DAPI) (Sigma-Aldrich, D9542). The following primary antibodies were used: anti-NEK1 (1:100, sc-398813, Santa Cruz Biotechnology), anti-NEK1-c-terminal (1:100, PA5-15336, Thermo Scientific), anti-adenylyl cyclase type 3 (ACIII) (1:200, ab125093, Abcam), anti-acetylated α-tubulin (1:200, T6793, Sigma-Aldrich), Alexa Fluor® 647 Anti-alpha Tubulin (1:1000, ab190573, Abcam), anti-ARL13B (1:200, 17711-1-AP, Proteintech), anti-smoothened (Smo) (1:200, ab72130, Abcam), anti-phospho-Aurora A (T288) (1:200, MA5-14904, Invitrogen), anti-Pericentrin (1:200, ab228144, Abcam), anti-γ-tubulin (1:200, T65557, Sigma-Aldrich), anti-TOM20 (1:200, ab186734, Abcam), anti-phospho-γH2AX (S139) (1:500, ab26350, Abcam), anti-islet1/2 (1:500, 39.4D5, Developmental Studies Hybridoma Bank), anti-TUJ1 (1:20000, PRB-435P, Biolegend), anti-ACIII (1:200, MCA-1 A12, EnCor Biotechnology), anti-MAP2 (1:200, ab5392, Abcam), anti-SMI-32 (1:200, 2937, Cell Signaling Technology), and anti-cleaved caspase-3 (1:200, 9661s, Cell Signaling Technology). Secondary antibodies included Alexa Fluor 488-, 555-, and 633-conjugated antibodies (1:500; A11001, A11008, A21422, A21428, and A21082, respectively; Invitrogen). Images were acquired with a Leica TCS SP8 laser-scanning confocal microscope (Leica) using an HC PL APO CS2 63x/1.40 objective.

### Image analysis

Cilia were counted in approximately >100 cells for each experimental condition from three experiments. The percentage of ciliated cells was calculated as follows: (total number of cilia/total number of nuclei in each image) × 100. Cilia lengths were measured with ImageJ (NIH) [44], and the average cilium length was calculated. For fluorescence intensity quantification, images were taken using the same settings in the same experiment and then measured using Image J. Briefly, the fluorescence signal (pixel area) around the basal body was selected using a tool (circle), and the integrated density (mean gray value) of the area was measured. Similarly, in the same field, an area with no fluorescence signal (next to a cell) was measured for the background fluorescence intensity. The corrected fluorescence was calculated using the following formula: corrected fluorescence = integrated density − (area of the pixel with signal × mean background fluorescence intensity). Mitochondrial lengths (in μm) obtained in the skeletonized mitochondrial network were measured using the Mitochondria Analyzer plugin in Image J, and the average mitochondrial length was calculated.

### Mitochondrial transmembrane potential analysis

The mitochondrial membrane potential (ΔΨ_m_) was assessed in live primary fibroblasts using membrane-permeant JC-1 dye following the manufacturer’s instructions (Sigma-Aldrich). Briefly, fibroblasts were washed and incubated with 5 μg/ml JC-1 dye for 20 min at 37 °C. The cells were then rinsed with culture medium, and their images were obtained using the Applied Precision DeltaVision fluorescence microscopy system (GE Healthcare, Chicago, IL, USA). Fluorescent intensity per cell in four image frames for each group was quantified using ImageJ. Mitochondrial membrane potential in each group was quantified by calculating the ratio of red (JC-1 monomers) to green (JC-1 aggregates) fluorescent intensity and normalized to the control (red-to-green fluorescent ratio of control was considered as 1).

### Transmission electron microscopy

Human fibroblasts were grown in 35-mm glass-bottomed culture dishes to 50%–60% confluency. The cells were then fixed with 2 ml of a fixative solution containing 2% paraformaldehyde (EM-grade, EMS) and 2.5% glutaraldehyde (GA, EMS) diluted in sodium cacodylate buffer. After washing, then post-fixed in 2% osmium tetroxide (OsO4) containing 1.5% potassium ferrocyanide for 1 h at 4 °C. The fixed cells were dehydrated by incubation in an ethanol series (50%, 60%, 70%, 80%, 90%, and 100%) for 10 min at each concentration and mounted in embedding medium. Then, 60-nm sections were cut horizontally in the plane of the block (UC7; Leica Microsystems, Germany) and mounted on copper slot grids with a specimen support film. Then, the sections were double-stained with 2% uranyl acetate for 10 min and lead citrate for 5 min. The sections were then observed using a Tecnai G2 transmission electron microscope at 120 kV (Thermo Fisher, USA). Data for abnormal mitochondrial structure were analysed using Image J software.

### HDAC6 activity

A fluorogenic HDAC6 assay kit (#50076; BPS Bioscience, San Diego, CA, USA) was used to determine the HDAC6 activity following the manufacturer’s instructions. Briefly, the fibroblast cell lysates were diluted in the HDAC assay buffer and mixed with the substrate. The HDAC developer was added, and the cells were subsequently incubated with the cell lysates. A Tecan Infinite M Nano Plus plate reader was used to measure the fluorescence intensity at excitation and emission wavelengths of 360 and 460 nm, respectively.

### Measuring the intracellular Ca^2+^

A calcium assay kit (#ab102505; Abcam, Cambridge, MA, USA) was used to measure the intracellular Ca^2+^, according to the manufacturer’s instructions. Briefly, the cells were harvested and homogenized in calcium assay buffer after 48 h of fibroblast incubation in the absence of serum. To each well of a 96-well plate, 50 µL of cell lysate was added. To each well, 90 µL of the chromogenic reagent was added followed by 60 µL of the calcium assay buffer. The samples were mixed and incubated at RT for 10 min in the dark. Standard curves were generated using standard dilutions. The absorbance was recorded at 575 nm using a Tecan Infinite M Nano Plus microplate reader.

### Measuring the cytosolic Ca^2+^ using Fluo-3/AM probes

Fibroblasts were stained with 1 mM Fluo3-AM (# 39294; Sigma) for 30 min at 37 °C in the dark incubator. The cells were subsequently washed with Ca^2+^-free Tyrode’s solution to remove the residual dye. A Leica TCS SP8 laser scanning confocal microscope (Leica) was used to measure the green fluorescence of Ca^2+^. Excitation and emission wavelengths were 488 and 530 nm, respectively.

### Statistical analysis

Data are presented as mean ± standard errors of the mean. Comparisons were made using the Student’s *t*-test or one-way analysis of variance (ANOVA) with post-hoc Tukey’s tests, using GraphPad Prism 10. Statistical significance was set at *p*-value < 0.05.

### Data availability

Data supporting the results of this study can be obtained from the corresponding author upon request.

## Results

### Characterizing *NEK1* variants in patients with sporadic ALS

**Fig. 1:**
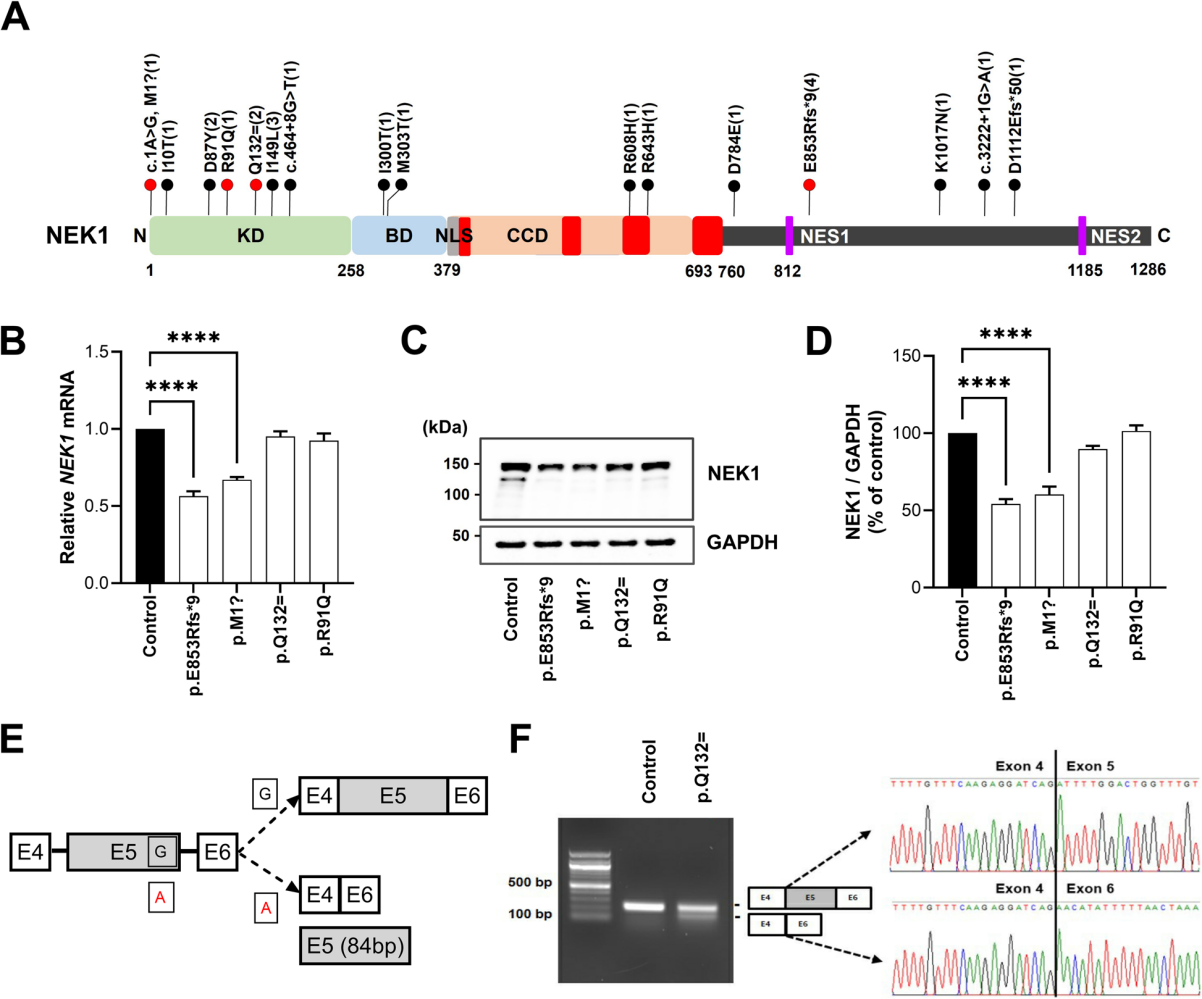
Identifying *NEK1* variants in patients with ALS by exome sequencing. **A** Schematic representation of the NEK1’s domain structure exhibiting the kinase domain (KD), basic domain (BD), four coiled-coil domains (CCD), nuclear localization signal (NLS), and two nuclear export sequences (NES). The black text indicates each domain’s amino acid (a.a) position. Upward lollipops (black), shown in bold, indicate the variants identified; lollipops (red) indicate the variants from which skin fibroblasts were obtained. Numbers in parentheses indicate the number of affected individuals. Amino acids in the figure are indicated with the one-letter code instead of the three-letter code. **B** Relative *NEK1* mRNA levels in the control and patient fibroblasts. Data represent mean ± standard errors of the mean (SEM) (from four independent experiments). Comparisons were made against the control (****P < 0.0001; one-way analysis of variance (ANOVA) with post-hoc Tukey’s tests). **C** Western blot analysis of NEK1 in the cell lysates from the control and patient fibroblasts. GAPDH was used as a loading control. **D** Quantification of the normalized NEK1 protein expression from three independent experiments. Data represent mean ± SEM. Comparisons were made against the control (****P < 0.0001; one-way ANOVA with post hoc Tukey’s tests). **E** Schematic representation of the wild-type and mutant (c.396G>A, p.Q132=) sequences. Boxes indicate the exons (E4, E5, and E6) and lines indicate introns. The sequence surrounding the exon 5 donor splice site of the wild-type G and mutant A is indicated. The diagonal dashed lines represent two possible splicing patterns (E5 inclusion or skipping), and the two possible resulting splice products are shown schematically on the right panel. **F ***NEK1* exon 5 splicing assay. Total RNA extracted from the control and patient fibroblasts carrying the p.Q132= variant was analyzed via reverse transcription polymerase chain reaction to detect inclusion or skipping of exon 5 (see schematic diagrams in **E**). The c.396G>A variant produced two bands in the gel images. The smaller band corresponds to the aberrant splicing of exon 5, resulting in complete exon skipping, as confirmed through Sanger sequencing. Sequence chromatograms illustrate the read-through at each exon junction, and sequence alignment indicates exon 5 deletion

We studied *NEK1*’s genetic contribution to ALS pathogenesis by analyzing the WES data from 920 Korean patients with ALS and identifying 16 *NEK1* variants in 23 patients (23/920, 2.5%), including two novel frameshift variants (p.E853Rfs*9, *n*=4; p.D1112Efs*50, *n*=1), one initiation codon variant (p.M1?, *n*=1), one splicing variant of the consensus splice site (c.3222+1G>A, *n*=1), one synonymous splicing variant (p.Q132=, *n*=2), two splicing variants, and ten missense variants (Fig. 1A and Table S1). Table S1 lists all identified *NEK1* variants. None of the patients with *NEK1* variants had a family history. Table S2 summarizes the demographic and clinical characteristics of the 23 patients with sALS *NEK1* variants. We identified p.D1112Efs*50 as a likely pathogenic variant, whereas the other variants were classified as variants of uncertain significance according to the ACMG/AMP guidelines [40]. Additional variants, such as p.Met1?, which removed the first methionine to initiate translation, has been repeatedly identified in Europe [11, 14] and one missense variant, p.R608H, has been previously reported in Taiwan [10]. The nucleotide change in the synonymous variant c.396G>A (p.Q132=) is located at the end of exon 4. Additionally, the variant was absent from the population database, revealing the possibility of aberrant splicing in the in silico analysis using SpliceAI.

We studied the contribution of these variants in ALS by performing a series of functional studies on four patients with *NEK1* variants available for skin biopsies, including one frameshift (p.E853Rfs*9), one p.M1?, one synonymous splicing variant (p.Q132=), and one missense variant (p.R91Q). We first examined the mRNA and protein expression levels of *NEK1* in available patient-derived fibroblasts (p.E853Rfs*9, p.M1?, p.R91Q, and p.Q132=). Compared with the control fibroblasts, *NEK1* mRNA and protein levels were reduced by approximately 50% in patient fibroblasts carrying p.E853Rfs*9 and p.M1? variants, using qPCR and western blotting analyses (Fig. 1B–D). We expected two *NEK1* variants, p.E853Rfs*9 and p.M1?, to result in the LOF of *NEK1* at the mRNA and protein levels by nonsense-mediated decay and loss of the translation start codon, respectively. Additionally, the c.396G>A (p.Q132=) variant was expected to skip exon 5 as predicted by the splice-site in silico software, leading to a putative aberrant protein product (Fig. 1E). We performed transcript analysis using patient fibroblasts to identify the splicing impact of the c.396G>A (p.Q132=) variant. Patient fibroblasts carrying the c.396G>A variant exhibited two bands as observed using RT-PCR, with the aberrant isoform lacking exon 5 derived from the mutated allele. The deletion of exon 5 (84 bp) by Sanger sequencing of the PCR products was confirmed (Fig. 1F), which was validated using SpliceAI.

### ALS-linked *NEK1* variants impaired primary cilia formation and Shh signaling

**Fig. 2:**
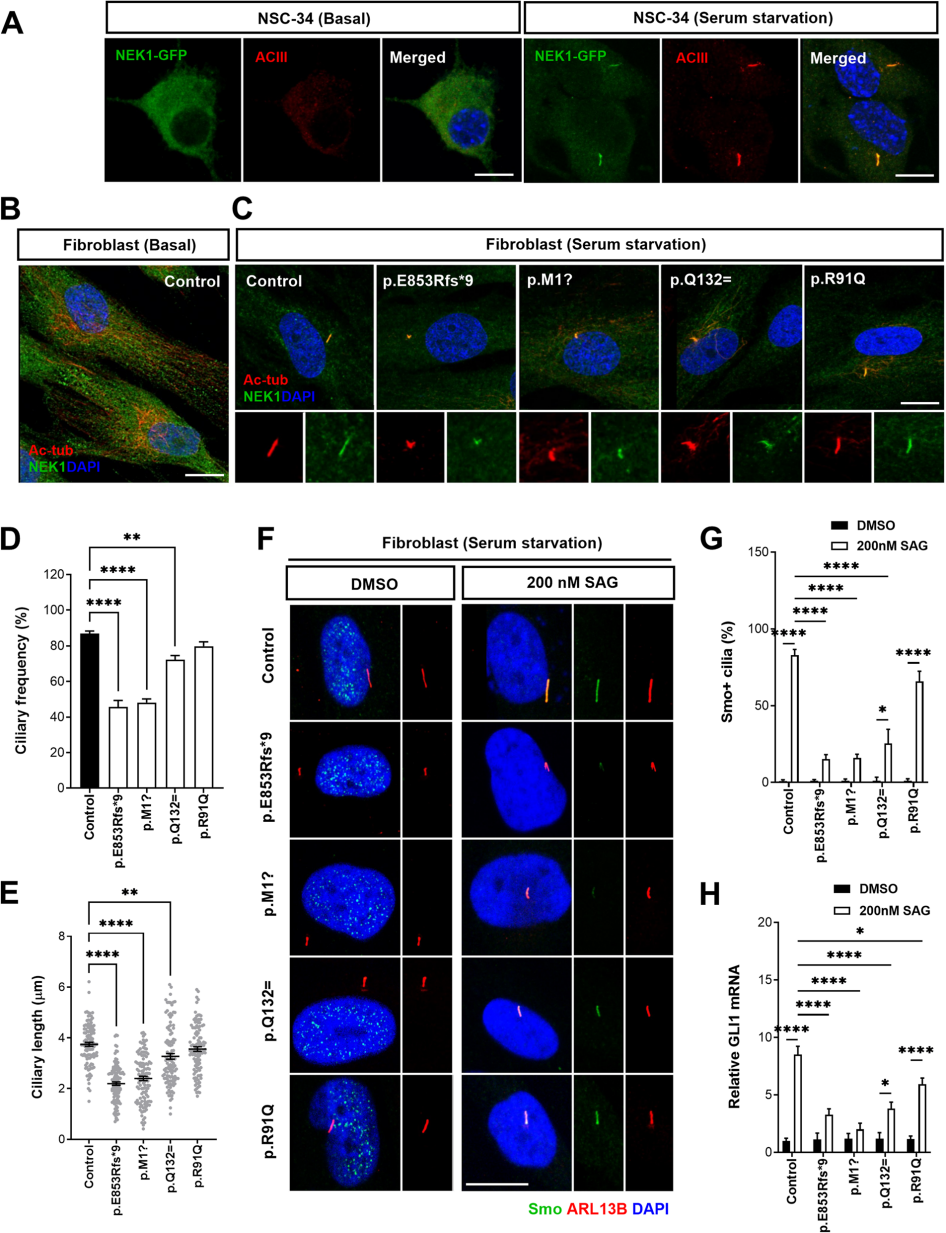
ALS-linked *NEK1* variants perturb primary ciliogenesis and Shh signaling in patient fibroblasts. **A** Subcellular distribution of GFP-tagged NEK1 WT in transfected NSC-34 cells under basal culture conditions (left panel) or serum starvation for 48 h (right panel). Fluorescence images of the primary cilia in the transfected NSC-34 cells. Cells were stained with ACIII (red, neuronal cilia marker). Nuclei were stained with DAPI. Scale bar: 10 µm. **B** Representative fluorescence images of endogenous NEK1 (green) and acetylated α-tubulin (red, ciliary axoneme marker) in control fibroblasts under basal culture conditions. Nuclei were stained with DAPI. Scale bar: 10 µm. **C** Representative fluorescence images of endogenous NEK1 (green) and acetylated α-tubulin (red, ciliary axoneme marker) for primary cilia formation in control and patient fibroblasts stimulated with serum starvation for 48 h. The bottom panels indicate higher magnification views of the primary ciliary regions. Nuclei were stained with DAPI. Scale bar: 10 µm. **D**-**E** Quantification of the ciliary frequency (**D**) and the ciliary length (**E**) in **C**. The >100 cells per condition were quantified per replicate experiment (*n* = 3). Data represent mean ± SEM. Comparisons were made against the control (**P < 0.01, ****P < 0.0001; one-way ANOVA with post-hoc Tukey’s tests). **F** Representative fluorescence images of Smo (green) and ARL13B (red, cilia marker) in the control and patient fibroblasts stimulated with serum starvation for 48 h. We examined the Smo translocation to the cilium in response to the Shh ligand-mediated signaling in the fibroblasts treated with DMSO or 200 nM Smo agonist (SAG) for 24 h. The right panels illustrate higher magnification views of the primary ciliary regions. Nuclei were stained with DAPI. Scale bar: 10 µm. **G** Quantification of Smo^+^ cells frequency in F. The >100 cells per condition were quantified per replicate experiment (*n* = 3). Data represent mean ± SEM. Comparisons were made against the DMSO-treated fibroblast (*P < 0.05, ****P < 0.0001; one-way ANOVA with post-hoc Tukey’s tests). **H** Relative changes in the *GLI1* mRNA levels in the control and patient fibroblasts treated with DMSO or 200 nM SAG for 24 h. Data represent mean ± SEM (from three independent experiments). Comparisons were made against the DMSO-treated fibroblasts (*P < 0.05, ****P < 0.0001; one-way ANOVA with post-hoc Tukey’s tests)

NEK1 is involved in primary cilia formation and is localized in the basal body region and ciliary axoneme [45, 46]. We confirmed these findings by examining NEK1’s subcellular distribution into the NSC-34 motor neuronal cells. GFP-tagged NEK1 was transfected into the NSC-34 cells, and NEK1 distribution was observed under basal culture conditions or serum starvation to induce ciliogenesis. In the cultured cells, the ciliary assembly process is typically triggered by serum starvation to induce cell cycle arrest in the G0/G1 phase while restimulation with serum results in cell cycle re-entry and ciliary disassembly [20, 47]. Primary cilia in the NSC-34 cells were labeled with the ACIII antibody, a marker for neuronal primary cilia [29]. Under basal conditions, NEK1 was primarily expressed in the cytoplasm, whereas after serum starvation, it was observed in a typical cilium structure (Fig. 2A). The *NEK1* variants found in the ALS cohort were hypothesized to disrupt NEK1’s role in primary ciliary structure and function.

We determined the subcellular distribution of endogenous NEK1 in patient fibroblasts under basal or serum-starvation culture conditions to investigate whether ALS-associated variants of *NEK1* regulate ciliogenesis. The frequency and length of the primary cilia were quantified by immunostaining with NEK1 and acetylated α-tubulin (ciliary axoneme marker) antibodies. Under basal conditions, NEK1 exhibited a predominantly cytoplasmic distribution with tubule-like structures in the control fibroblasts (Fig. 2B, Fig. S1). NEK1 was mainly localized in the primary cilia of the control and patient fibroblasts carrying ALS-linked variants after inducing ciliary formation by serum starvation (Fig. 2C). However, the length and frequency of the primary cilia were significantly reduced in the patient fibroblasts carrying p.E853Rfs*9, p.M1?, or p.Q132= variants compared to the control fibroblasts (*P* < 0.001) (Fig. 2C–E). Particularly, patient fibroblasts carrying *NEK1* LOF variants (p.E853Rfs*9 or p.M1?) exhibited severely abnormal ciliary morphology, such as predominantly shortened cilia or branched, often tapered, and twisted cilia (Fig. 2C, Fig. S2A-B). Contrastingly, the p.R91Q missense variant exhibited normal ciliogenesis, implying that this missense variant had less effect on primary ciliogenesis (Fig. 2C–E). We confirmed these findings in motor neuron-like cells by assessing whether *NEK1* variants regulate the ciliary structure. NSC-34 cells were transfected with GFP-tagged NEK1 constructs, and the ciliary length was measured after serum starvation using the endogenous neuronal ciliary marker ACIII. NEK1-WT and other variants were diffusely localized in the cytoplasm and accumulated in the cilia after serum starvation. Furthermore, the splicing variant (p.Q132=)-expressing NSC-34 cells exhibited reduced ciliary length and frequency compared to the NEK1-WT. In contrast, p.R91Q demonstrated ciliogenesis similar to that of the NEK1-WT (Fig. S3A-C). These data suggest that aberrant expression of NEK1 causes morphological defects in primary cilia.

Primary cilia serve as hubs for several signaling pathways, of which the Shh signaling pathway is prominent in the brain [18, 19, 21, 48]. The Shh signaling affects neurogenesis and neural patterning during central nervous system (CNS) development, and its dysregulation in the brain contributes to neurodegenerative diseases [21, 49]. Therefore, we further investigated whether an impaired ciliary morphology in patient fibroblasts carrying ALS-linked *NEK1* variants induces Shh signaling alterations. We measured the responses to stimulation with a smoothened agonist (SAG) to investigate defects in Shh signaling. In response to 200 nM SAG treatment for 24 h, we observed that Smoothened (Smo) co-localized with ADP ribosylation factors-like GTPase 13b (ARL13B), a ciliary marker protein, in the control fibroblasts. However, patient fibroblasts carrying *NEK1* LOF variants lacked Smo expression within the ciliary axoneme (Fig. 2F and G). Under the same conditions, the p.Q132= variant with shortened cilia also caused reduced Smo accumulation in the ciliary axoneme. Subsequently, we assessed the mRNA levels of the transcription factor *GLI1*, which activates Shh signaling downstream of Smo, using qPCR analysis. The *GLI1* mRNA levels are known to rapidly increase upon Shh activation [50, 51] and we confirmed similar results in the control fibroblasts. Consistent with ciliary defects, the *GLI1* mRNA expression induced by SAG treatment was inhibited in patient fibroblasts carrying *NEK1* LOF variants compared to the control fibroblasts. This indicated that Shh signaling was disrupted (Fig. 2H). Contrastingly, the p.Q132= variant exhibited mildly increased *GLI* mRNA levels upon SAG treatment; however, the increase was insufficient compared to that in the control fibroblasts. The p.R91Q missense variant demonstrated a similar response to Shh signaling as the control (Fig. 2F–H). These data suggest that ALS-linked variants of *NEK1* may perturb ciliary assembly and disrupt Shh signal transduction. Additionally, we also analyzed the expression of ciliogenesis-related genes that are associated with ciliopathy or previously known to be dysregulated in PD state [52, 53]. Among them, we found that the expressions of some cilia-associated genes, such as intraflagellar transport genes (*IFT80*, *IFT20, DYNC2H1*), BBSome genes (*BBS1, BBS2, ARL6*), ciliogenesis genes (*OFD1, OCRL*), and axoneme genes (*TUBA1A, TUBB*) were downregulated (Fig. S2C). These data support that ALS-linked variants of *NEK1* may disrupt ciliogenesis by regulating the expression of ciliary genes.

### ALS-linked *NEK1* variants alter cell cycle progression and induce ciliary disassembly

**Fig. 3:**
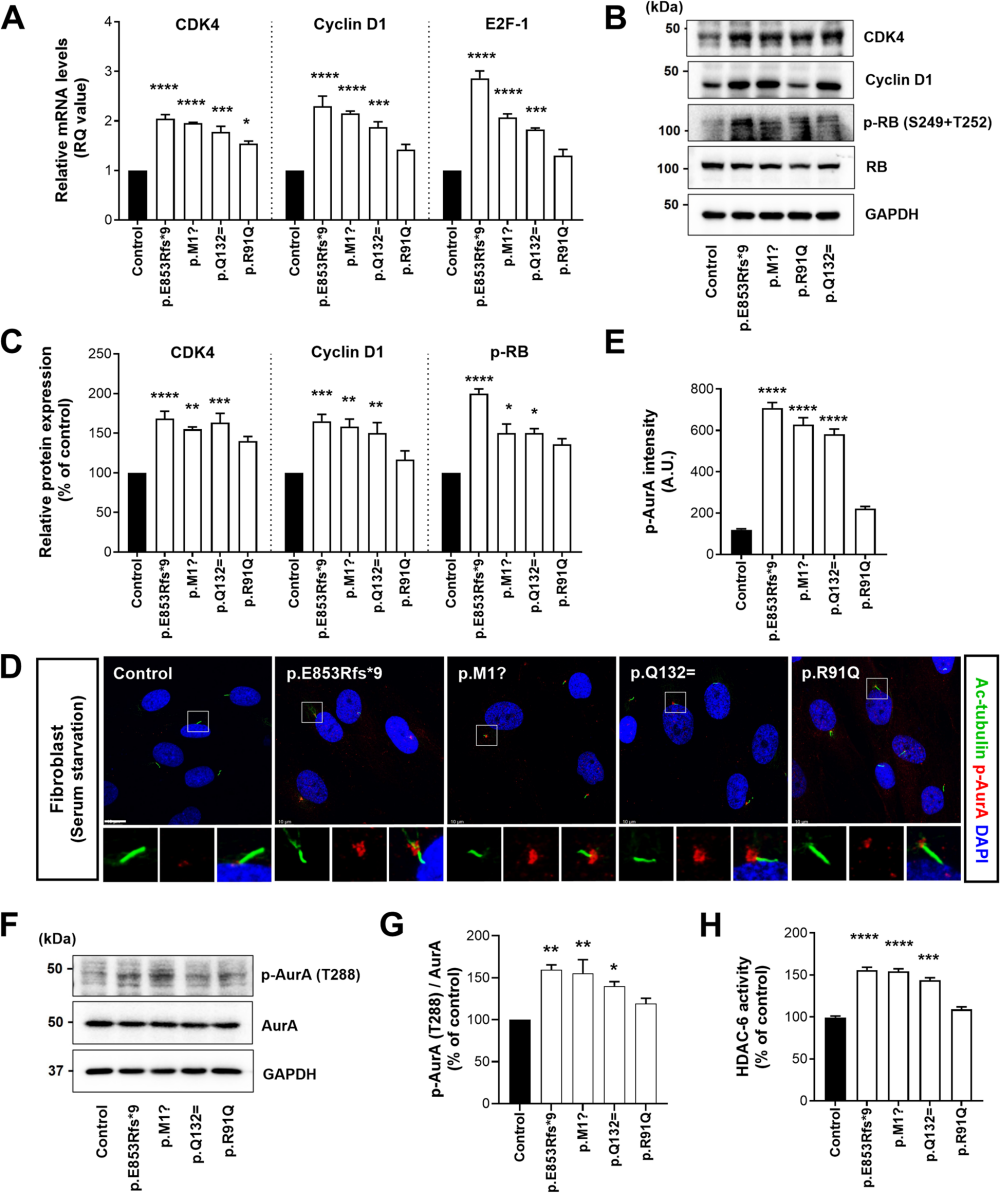
ALS-linked *NEK1* variants demonstrate aberrant cell cycle progression and activated ciliary disassembly axis in patient fibroblasts. **A** Relative mRNA levels of the cell cycle regulators (*CDK4*, *Cyclin D1*, and *E2F-1*) from G1 to S phase in the control and patient fibroblasts stimulated with serum starvation for 48 h. Data represent mean ± SEM (from three independent experiments). Comparisons were made against the control (*P < 0.05, ***P < 0.001, ****P < 0.0001; one-way ANOVA with post-hoc Tukey’s test). **B** Western blot analysis of the control and patient fibroblasts stimulated with serum starvation for 48 h using anti-CDK4, anti-Cyclin D1, anti-p-RB (S249+T252), and anti-RB antibodies. GAPDH was used as a loading control. **C** Quantification of the normalized CDK4, Cyclin D1, and p-RB (S249+T252) protein expression from three independent experiments. CDK4 and Cyclin D1 intensities were normalized to GAPDH. p-RB (S249+T252) intensities were normalized to total RB. Data represent mean ± SEM.Comparisons were made against the control (*P < 0.05, **P < 0.01, ***P < 0.001, ****P < 0.0001; one-way ANOVA with post-hoc Tukey’s tests). **D** Representative fluorescence images of the activated AurA (p-AurA) in control and patient fibroblasts stimulated with serum starvation for 48 h. Cells were stained with p-AurA (phosphorylated T288) (red), acetylated α-tubulin (green, ciliary axoneme marker), and DAPI (blue). Scale bar: 10 µm. The lower panels illustrate higher magnification views of the cilia. **E** Quantification of p-AurA (red) intensity at the ciliary base described in **D**. The >100 cells per condition were quantified per replicate experiment (*n* = 3). Data represent mean ± SEM. Comparisons were made against the control (****P < 0.0001; one-way ANOVA with post-hoc Tukey’s test). **F** Western blot analysis of the control and patient fibroblasts stimulated with serum starvation for 48 h using anti-phospho-AurA (T288) and anti-AurA antibodies. GAPDH was used as a loading control. **G** Quantification of the normalized p-AurA protein expression from three independent experiments. p-AurA intensity was normalized to total AurA. Data represent mean ± SEM.Comparisons were made against the control (*P < 0.05, **P < 0.01; one-way ANOVA with post-hoc Tukey’s tests). **H** Quantification of the HDAC6 activity in the control and patient fibroblasts stimulated with serum starvation for 48 h from three independent experiments. Data represent mean ± SEM.Comparisons were made against the control (***P < 0.001, ****P < 0.0001; one-way ANOVA with post-hoc Tukey’s test)

The regulation of primary cilia assembly and disassembly is tightly coordinated with cell cycle exit and re-entry. Animal models and postmortem examinations of the human CNS have revealed aberrant cell cycle re-entry into the apoptotic neurons [54]. Injured neurons exhibit DNA replication, increased expression of the cell cycle markers, including cyclins, cyclin-dependent kinases (CDKs), and phosphorylation of the retinoblastoma protein (RB), which serves as a master switch for cell cycle re-entry. We analyzed the mRNA levels of cell cycle regulators (*CDK4*, *Cyclin D1*, and *E2F-1*) from the G1 to S phase in patient and control fibroblasts to investigate the association between ciliary dysfunction by *NEK1* variants with cell cycle regulation. We observed increased mRNA levels of the cell cycle regulators (*CDK4*, *Cyclin D1*, and *E2F-1*) in patient fibroblasts carrying *NEK1* variants compared to those in the control fibroblasts (Fig. 3A). Additionally, patient fibroblasts exhibited increased expression of the cell cycle regulatory proteins in the G1–S phase, including CDK4, Cyclin D1, and p-RB by western blotting analysis (Fig. 3B and C). The p.R91Q variant exhibited a lesser impact on cell cycle progression (Fig. 3A-C). These results suggest that changes in cell cycle progression are associated with the resorption of primary cilia induced by ALS-linked *NEK1* variants.

The core players in ciliary disassembly during cell cycle re-entry are represented by the Aurora A (AurA)-HDAC6 axis [55]. AurA kinase at the basal body of cilia induces HDAC6 phosphorylation and activation, a cytoplasmic tubulin deacetylase that promotes ciliary disassembly. We examined the AurA and HDAC6 activities in the patient and control fibroblasts to evaluate the changes in the ciliary disassembly axis due to *NEK1* mutations. In the control fibroblasts, AurA activation (by T288 phosphorylation) was barely detectable in the ciliary basal bodies under serum-starved conditions (Fig. 3D and E). However, patient fibroblasts carrying *NEK1* variants, except p.R91Q, exhibited markedly increased AurA levels, mostly in the shortened ciliary cells (Fig. 3D and E). Similarly, AurA activation in the patient fibroblasts was confirmed using western blot analysis (Fig. 3F and G). HDAC6 is an important downstream effector of AurA in ciliary disassembly and requires intact deacetylation activity [55]. We analyzed HDAC6 deacetylation activity under serum starvation conditions to confirm HDAC6 activation by phosphorylating AurA in the *NEK1* variants. Patient fibroblasts carrying *NEK1* variants, except p.R91Q, exhibited increased HDAC6 activity compared to the control fibroblasts (Fig. 3H). These results indicate that ALS-linked *NEK1* variants induce the activation of the AurA- HDAC6 axis, leading to ciliary disassembly.

### ALS-linked *NEK1* variants perturb Ca^2+^-dependent regulation of primary ciliogenesis

**Fig. 4:**
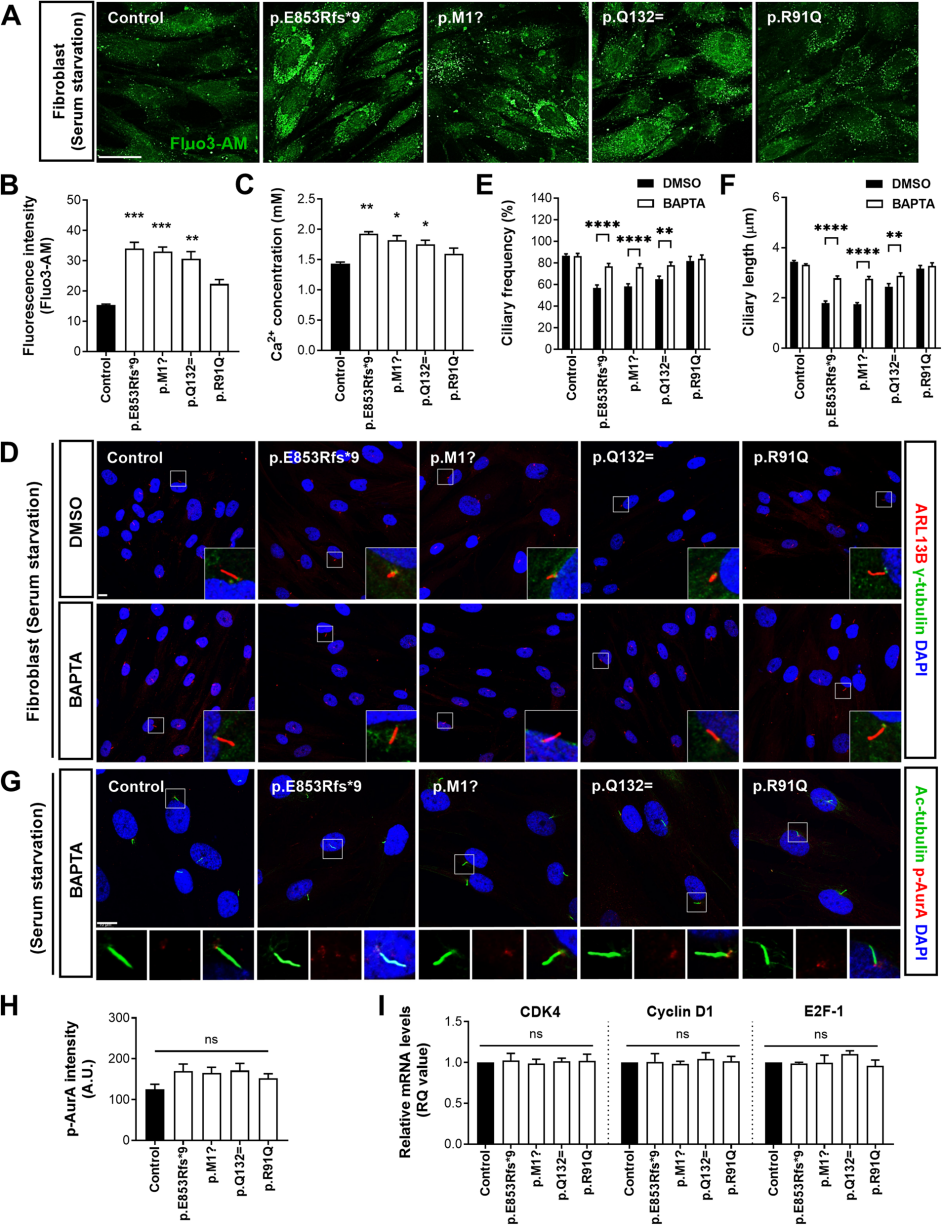
ALS-linked *NEK1* variants impair intracellular Ca^2+^ homeostasis and regulate cilia in a Ca^2+^-dependent manner. **A** Representative images of the cytosolic Ca^2+^ in the control and patient fibroblasts stimulated with serum starvation for 48 h. Fluo3-AM, a calcium indicator with green fluorescence, was visualized using confocal microscopy. Scale bar: 10 µm. **B** Fluorescence intensities of Fluo 3-AM images (**A**) of cytosolic Ca^2+^ were quantified using ImageJ with low-power field images. Data represent mean ± SEM (from three independent experiments). Comparisons were made against the control (**P < 0.01, ***P < 0.001; one-way ANOVA with post-hoc Tukey’s test). **C** Quantitative analysis of the intracellular Ca^2+^ concentration in the control and patient fibroblasts stimulated with serum starvation for 48 h. Data represent mean ± SEM (from three independent experiments). Comparisons were made against the control (*P < 0.05, **P < 0.01; one-way ANOVA with post-hoc Tukey’s test). **D** Representative fluorescence images of the primary cilia in the starved control and patient fibroblasts treated with DMSO or 10 µM of the Ca^2+^ chelator BAPTA for 60 min. The cilia and basal bodies were visualized with antibodies against ARL13B (red) and γ-tubulin (green), respectively. The nuclei were stained with DAPI (blue). The right panels exhibit higher magnification views of the cilia and basal body. Scale bar: 10 µm. **E**-**F** Quantification of the ciliary frequency (**E**) and ciliary length (**F**) in D. The >100 cells per condition were quantified per replicate experiment (*n* = 3). Data represent mean ± SEM. Comparisons were made against the DMSO-treated fibroblasts (**P < 0.01, ****P < 0.0001; one-way ANOVA with post hoc Tukey’s tests). **G** Representative fluorescence images of activated AurA (p-AurA) in the starved control and patient fibroblasts treated with DMSO or 10 µM of the Ca^2+^ chelator BAPTA for 60 min. Cells were stained with p-AurA (phosphorylated T288) (red), acetylated α-tubulin (green, ciliary axoneme marker), and DAPI (blue). The lower panels demonstrate higher magnification views of the cilia. Scale bar: 10 µm. **H** Quantification of p-AurA (phosphorylated T288, red) intensity at the ciliary base described in G. The >100 cells per condition were quantified per replicate experiment (*n* = 3). Data represent mean ± SEM. Comparisons were made against the control (ns, not significant; one-way ANOVA with post-hoc Tukey’s test). **I** Relative mRNA levels of the cell cycle regulators (*CDK4*, *Cyclin D1*, and *E2F-1*) from G1 to S phase in control and patient fibroblasts pretreated with 10 µM BAPTA for 60 min and stimulated with serum starvation for 48 h. Data represent mean ± SEM (from three independent experiments). Comparisons were made against the control (ns, not significant; one-way ANOVA with post-hoc Tukey’s test)

AurA activation in ciliary disassembly requires interactions with Ca^2+^ and calmodulin (CaM), and Ca^2+^/CaM are important mediators of the ciliary disassembly process [56]. Dysregulation of intracellular Ca^2+^ homeostasis and excitotoxicity play vital roles in selective motor neuron vulnerability and degeneration in ALS [57, 58]. The intracellular Ca^2+^ levels were measured in the fibroblasts under serum starvation conditions using Fluo-3 AM, a fluorescent calcium indicator, and an intracellular Ca^2+^ concentration assay to investigate whether ALS-linked variants of *NEK1* are associated with impaired Ca^2+^ signaling. We observed that cytosolic Ca^2+^ levels (Fluo-3 fluorescence) were almost twofold as high in the patient fibroblasts carrying the p.E853Rfs*9, p.M1?, or p.Q132= variants compared to the control (Fig. 4A and B). Furthermore, the intracellular Ca^2+^ concentration was substantially elevated in the patient fibroblasts carrying LOF and *NEK1* splicing variants compared to that in the control (Fig. 4C), indicating the disruption of intracellular Ca^2+^ homeostasis.

Furthermore, we investigated the role of calcium in ciliary dynamics. We tested this by culturing fibroblasts with or without 1,2-bis(2-aminophenoxy)ethane-*N*,*N*,*N*′,*N*′-tetraacetic acid (BAPTA), a cell-penetrating intracellular Ca^2+^ chelator, to decrease intracellular free Ca^2+^. Pretreatment of cells with 10 µM BAPTA for 60 min restored ciliary defects induced by *NEK1* variants compared to the dimethyl sulfoxide (DMSO) treatment (Fig. 4D-F). Furthermore, the decrease in intracellular Ca^2+^ levels caused by BAPTA pretreatment in the *NEK1* variants reduced AurA activation to normal levels in the basal bodies under serum starvation (Fig. 4G and H). Additionally, BAPTA treatment normalized the increased mRNA levels of the cell cycle regulators (*CDK4*, *Cyclin D1*, and *E2F-1*) in patient fibroblasts carrying *NEK1* variants under serum starvation (Fig. 4I). These data suggest that ALS-linked *NEK1* variants perturb intracellular Ca^2+^ homeostasis and affect abnormal primary cilia formation via AurA activation in a Ca^2+^-dependent manner.

### ALS-linked *NEK1* variants perturb tubulin acetylation and mitochondrial dynamics

**Fig. 5:**
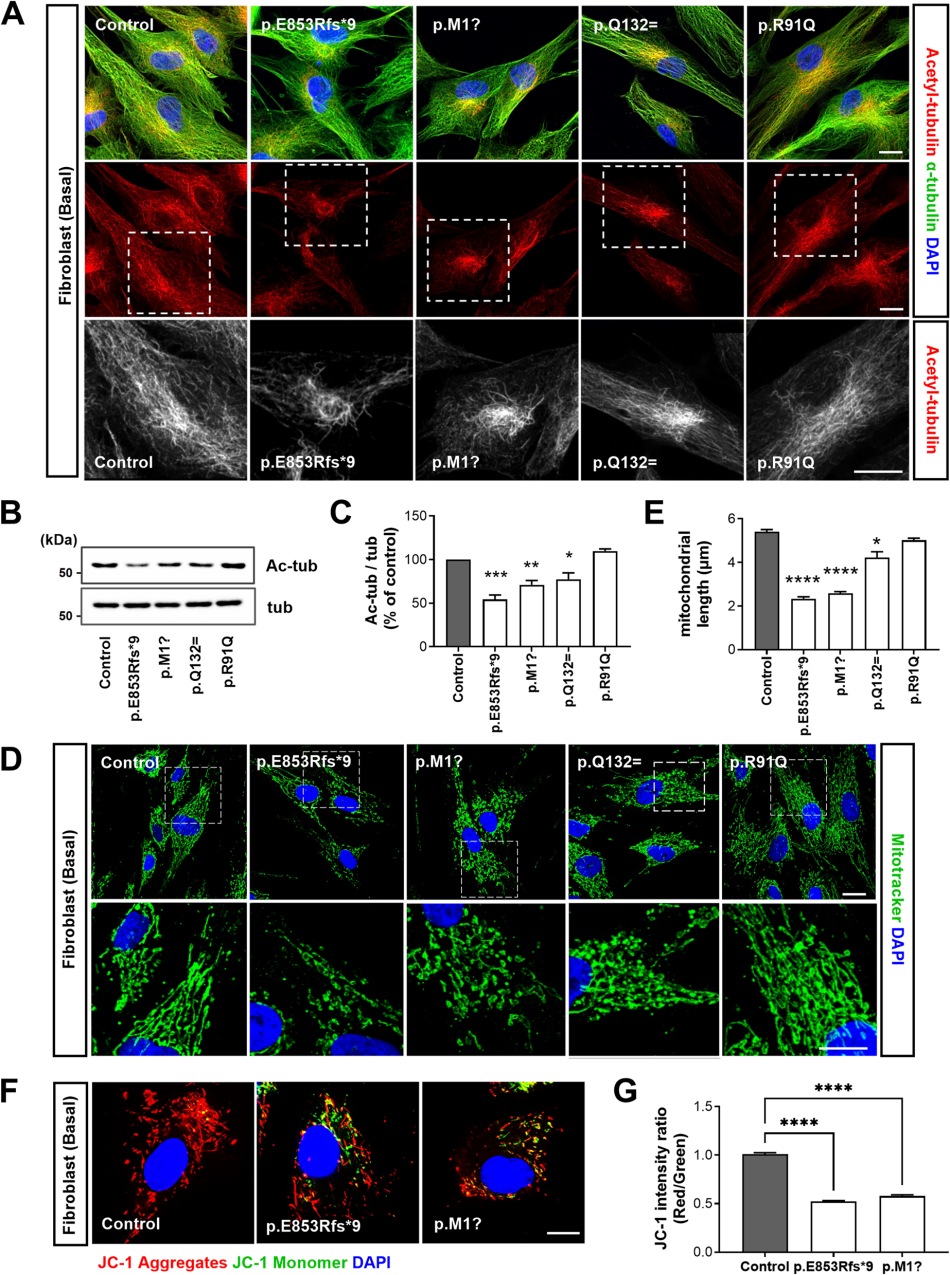
ALS-linked *NEK1* variants impaired tubulin acetylation and mitochondrial distribution in patient fibroblasts. **A** Representative fluorescence images of the acetylated α-tubulin (red) and α-tubulin (green) in control and patient fibroblasts under basal culture conditions. The bottom panels illustrate higher magnification views of white box regions. The nuclei were stained with DAPI. Scale bar: 10 µm. **B** Western blot analysis of the *NEK1* patient and control fibroblast lysates using anti-acetylated α-tubulin and anti-α-tubulin antibodies. **C** Quantification of normalized expression of acetylated α-tubulin (Ac-tub) from three independent experiments. Ac-tub intensities were normalized to total α-tubulin (tub). Data represent mean ± SEM. Comparisons were made against the control (*P < 0.05, **P < 0.01, ***P < 0.001; one-way ANOVA with post-hoc Tukey’s tests). **D** Representative fluorescence images of the mitochondria distribution in the control and patient fibroblasts labeled with MitoTracker Green FM (green). The bottom panels illustrate higher magnification views of the white box regions. The nuclei were stained with DAPI. Scale bar: 10 µm. **E** Quantification of the mitochondrial length in **D**. The >50 cells per condition were quantified per replicate experiment (*n* = 3). Data represent mean ± SEM. Comparisons were made against the control (*P < 0.05, ****P < 0.0001; one-way ANOVA with post-hoc Tukey’s test). **F** Fluorescence images of the mitochondrial membrane potential (Δψ_m_) using JC-1 dye-loaded control and *NEK1*-LOF patient fibroblasts. The abnormal accumulation of green-fluorescent JC-1 monomers in the mitochondria of *NEK1*-LOF patients but not control cells. Nuclei were stained with DAPI. Scale bar: 10 μm. **G** Mitochondrial membrane potential was quantified by analysis of the red-to-green fluorescence intensity ratio for the JC-1 probe. The >50 cells per condition were quantified per replicate experiment (*n* = 3). Data represent mean ± SEM. Comparisons were made against the control (****P < 0.0001; one-way ANOVA with post-hoc Tukey’s test)

Primary cilia are dynamic microtubule-based organelles, and impaired microtubule dynamics causes abnormal ciliogenesis [59]. Tubulin acetylation is the most frequent post-translational modification associated with stable microtubules [60]. We analyzed the microtubule state by immunostaining for acetylated α-tubulin in the patient and control fibroblasts under basal culture conditions to investigate whether ALS-linked variants of *NEK1* are associated with microtubule dynamics. Interestingly, acetylated α-tubulin exhibited weaker expression in the patient fibroblasts carrying the *NEK1* LOF variants (p.E853Rfs*9 and p.M1?) and the splicing variant (Q132=) compared to the control fibroblasts (Fig. 5A). Western blotting confirmed a reduction in acetylated α-tubulin in the patient-derived cells (Fig. 5B and C).

Impairment of the microtubule-based axonal transport is an early pathological mechanism in motor neuron diseases [61]. Particularly, regulating mitochondrial transport along the microtubule network is critical for neuronal function as mitochondria facilitate energy supply to the synaptic terminals. Abnormal microtubule dynamics affect the mitochondrial morphology and function [62]. Therefore, we investigated whether alteration of tubulin acetylation in patient fibroblasts paralleled abnormalities in mitochondrial morphology and function. Cells were stained with MitoTracker for mitochondrial labeling. In the control fibroblasts, MitoTracker Green signals appeared primarily as tubular networks extending throughout the cytoplasm (Fig. 5D). However, the LOF-variant fibroblasts (p.E853Rfs*9 and p.M1?) exhibited severe mitochondrial fragmentation and were more restricted to the perinuclear area than the control fibroblasts. The mitochondrial length was measured to quantify mitochondrial fragmentation. LOF-variant fibroblasts demonstrated a reduction in the average mitochondrial length compared to the control fibroblasts (Fig. 5E). Endogenous NEK1 was predominantly localized in the cytoplasm of the basal fibroblasts (Fig. 2B) and weakly expressed in tubule-like structures. We performed co-staining with TOM20 (a mitochondrial marker) and NEK1 in the fibroblasts to determine NEK1 localization in the mitochondria. NEK1 partially co-localized with the mitochondrial marker TOM20 (Fig. S4A) and detected in mitochondrial fractions from lysates of control and patient fibroblasts by western blotting analysis (Fig. S4B and C), these results suggesting that NEK1 affects mitochondrial function. Thus, we investigated whether mitochondrial abnormalities in the LOF-variant fibroblasts (p.E853Rfs*9 and p.M1?) are associated with mitochondrial dysfunction. We assessed mitochondrial membrane potential using JC-1, the cationic lipophilic dye. JC-1 accumulates as red fluorescent J-aggregates within the mitochondria at high membrane potentials and as green fluorescent monomers within the mitochondria at low membrane potentials. In living control fibroblasts loaded with JC-1, we observed strong red fluorescent signals but not green fluorescent signals (Fig. 5F and G), suggesting that the majority of mitochondria were functional. Contrastingly, green fluorescence signals were prominent in the JC-1-loaded LOF variant fibroblasts (p.E853Rfs*9 and p.M1?). Additionally, we examined whether the *NEK1* LOF mutations affected the mitochondrial cell death cascade by the loss of mitochondrial membrane potential, resulting in cytochrome c efflux from the mitochondrial intermembrane spaces. We separated cytosolic and mitochondrial fractions from the lysates of the control and patient fibroblasts and performed western blotting analysis of these fractions using anti-cytochrome c. Compared to the control fibroblasts, the LOF variants fibroblasts (p.E853Rfs*9 and p.M1?) exhibited substantially increased cytochrome c release from mitochondria into the cytosol (Fig. S4D and E). We also performed transmission electron microscopy (TEM) analysis of control and LOF variants fibroblasts (p.E853Rfs*9 and p.M1?) to examine the ultrastructural morphology. The LOF variants fibroblasts showed abnormal mitochondrial structure (without cristae structures and rupture of the outer mitochondrial membrane) (Fig. S4F and G). Thus, our study reveals that functional defects caused by ALS-linked *NEK1* variants induce abnormal tubulin acetylation and mitochondrial dysfunction.

### ALS-linked *NEK1* variants perturb the DNA damage response in patient-derived fibroblasts

**Fig. 6:**
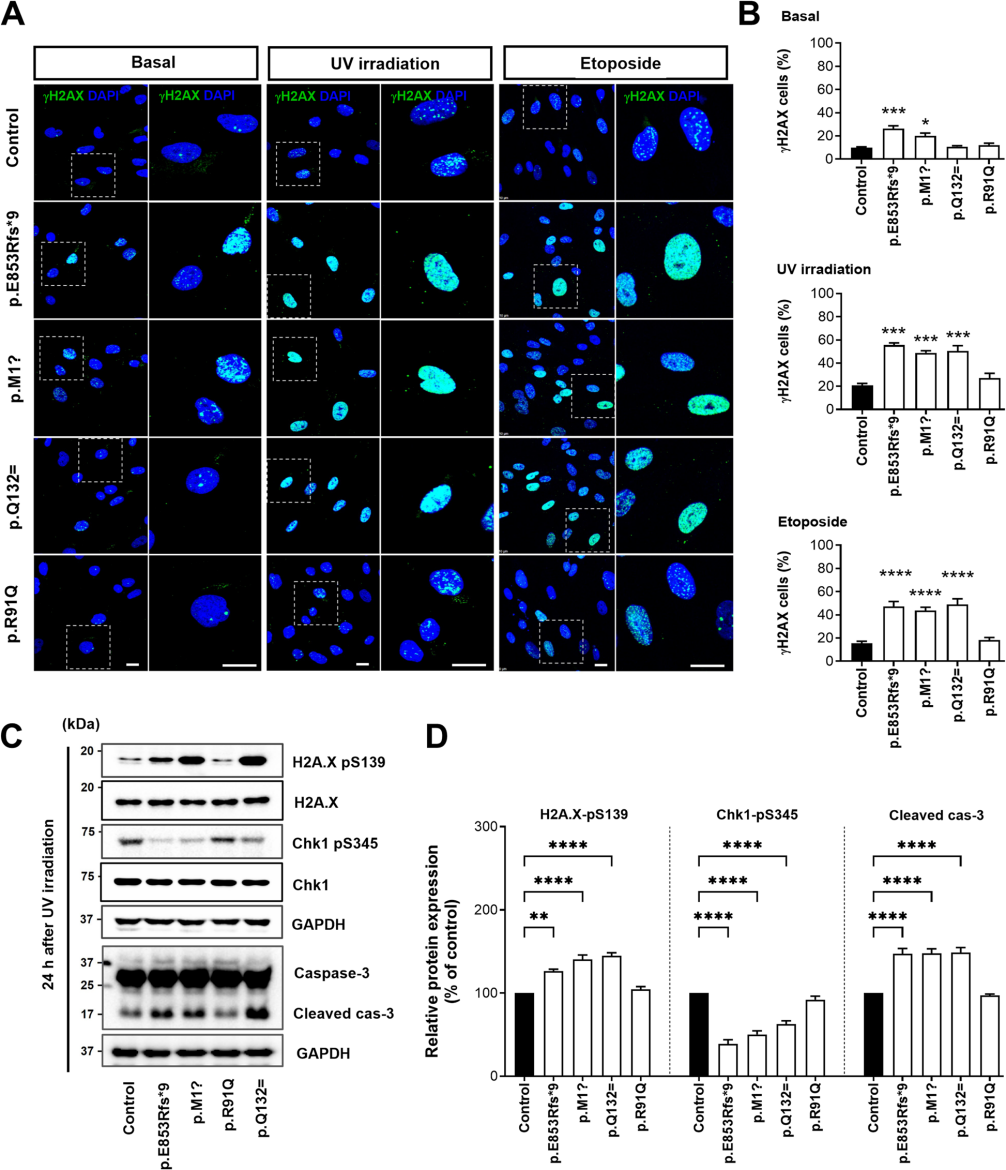
ALS-linked *NEK1* variants impaired DNA damage repair after ultraviolet (UV) irradiation or etoposide treatment in patient fibroblasts. **A** Representative fluorescence images of DNA damage response after UV irradiation or etoposide treatment in control and *NEK1* patient fibroblasts. Cells were stained with γH2AX (S139) (green, DNA damage marker) and DAPI (blue) either under basal condition, 24 h after 20 J/m^2^ UV irradiation, or 24 h after 20 μM etoposide treatment. The right panels illustrate higher magnification views of the white box regions. Scale bar: 10 µm. **B** Quantification of the γH2AX-positive cells in A. The >50 cells per condition were quantified per replicate experiment (*n* = 3). Data represent mean ± SEM. Comparisons were made against the control (*P < 0.05, ***P < 0.001, ****P < 0.0001; one-way ANOVA with post-hoc Tukey’s test). **C** Western blot analysis of the lysates from the control and *NEK1* patient fibroblasts at the time point of 24 h after UV irradiation using anti-γH2AX-pS139, anti-γH2AX, anti-Chk1-pS345, anti-Chk1, and anti-caspase-3 antibodies. GAPDH was used as a loading control. **D** Quantification of normalized expression of γH2AX-pS139, Chk1-pS345, and cleaved caspase-3 from three independent experiments.The band intensities of γH2AX-pS139 were normalized to the γH2AX intensity. The band intensities of Chk1-pS345 were normalized to the Chk1 intensity. The band intensities of cleaved caspase-3 (17 kDa) were normalized to the intensity of caspase-3 (35 kDa). Data represent mean ± SEM. Comparisons were made against the control (**P < 0.01, ****P < 0.0001; one-way ANOVA with post-hoc Tukey’s test)

The *NEK1* gene is known to play a role in DNA repair and DDR and contributes to neuronal death via these pathways in ALS [36, 63]. *NEK1*-ALS iPSC-MNs were previously shown to increase the γH2AX compared to the controls, suggesting that DNA damage is a feature of *NEK1*-associated ALS [36] γH2AX phosphorylation, as an indicator of double-strand breaks, occurs in nuclear foci following UV irradiation [64]. We examined γH2AX-positive foci accumulation in the fibroblasts at the basal level or after DNA damage by 20 J/m^2^ UV irradiation or 20 μM etoposide treatment to investigate whether ALS-linked *NEK1* variants are associated with alterations in DNA damage repair. In the *NEK1* LOF variant (p.E853Rfs*9) fibroblasts, the basal degrees of nuclear γH2AX were slightly increased compared to the control fibroblasts (Fig. 6A and B). After UV irradiation or etoposide treatment, the number of cells with γH2AX foci dramatically increased in the control fibroblasts, and most returned to basal levels after 24 h, as is previously well-known [65]. However, the *NEK1* LOF variants (p.E853Rfs*9 and p.M1?) and splicing variant (p.Q132=) fibroblasts still demonstrated a robust increased number of γH2AX-positive cells compared to the control fibroblasts (Fig. 6A and B), by failing to return to the basal level, indicating less efficient DDR after DNA damage. We examined the phospho-γH2AX expression in patient fibroblasts at 24 h after UV irradiation by western blotting and observed that γH2AX phosphorylation consistently increased in patient fibroblasts carrying p.E853Rfs*9, p.M1? and p.Q132= (Fig. 6C and D). DNA damage sensors detect DNA damage and activate master DNA repair kinases such as ataxia telangiectasia mutated (ATM) and ATM-and Rad3-related (ATR) signaling responses [66]. The repair kinases phosphorylate their downstream targets to induce cell cycle arrest through Chk1/Chk2 activation, p53 phosphorylation-mediated caspase-3 activation, and DNA repair. NEK1 is an essential ATR signaling regulator and is required to efficiently activate the checkpoint kinases ChK1/ChK2 within the ATM pathway [67]. We assessed pChk1 and caspase-3 levels in the control and patient fibroblasts following DNA damage induction to determine the effects of DNA damage signaling in the ALS-linked *NEK1* variants. At 24 h after UV irradiation, fibroblasts derived from the *NEK1* LOF variant (p.E853Rfs*9 and p.M1?) and the splicing variant (p.Q132=) exhibited a considerable decrease in pChk1 (S345) and an increase in cleaved caspase-3 compared to the control fibroblasts (Fig. 6C and D). We also analyzed the effect of DNA damage repair in NSC-34 cells expressing GFP-tagged NEK1 variants using UV irradiation to clarify whether aberrant expression of NEK1 is associated with alterations in DNA damage repair, leading to motor neuronal cell death. The expression level of anti-γH2AX phosphorylation after UV irradiation was markedly increased in the NSC-34 cells expressing NEK1-E853Rfs*9 or NEK1-Q132= compared to the transfected NEK1-WT (Fig. S5A and B). Next, cytotoxicity was assessed using cleaved caspase-3 as a marker of apoptotic cell death to further investigate whether *NEK1* variants affect motor neuronal cell survival. The NSC-34 cells were transfected with GFP-tagged NEK1-WT or ALS-linked *NEK1* variants and cultured for 24 h after UV irradiation. Compared with the NEK1-WT expressing cells, NSC-34 cells expressing NEK1-E853Rfs*9 or NEK1-Q132= exhibited substantially increased cleaved caspase-3 positive immunoreactivity (Fig. S5C and D). These results suggest that ALS-linked *NEK1*-LOF and its splice variants are impaired in response to DNA damage, leading to motor neuron cell death.

### Loss of *NEK1* function affects primary cilia formation, tubulin acetylation, mitochondrial distribution, and DNA damage repair

**Fig. 7:**
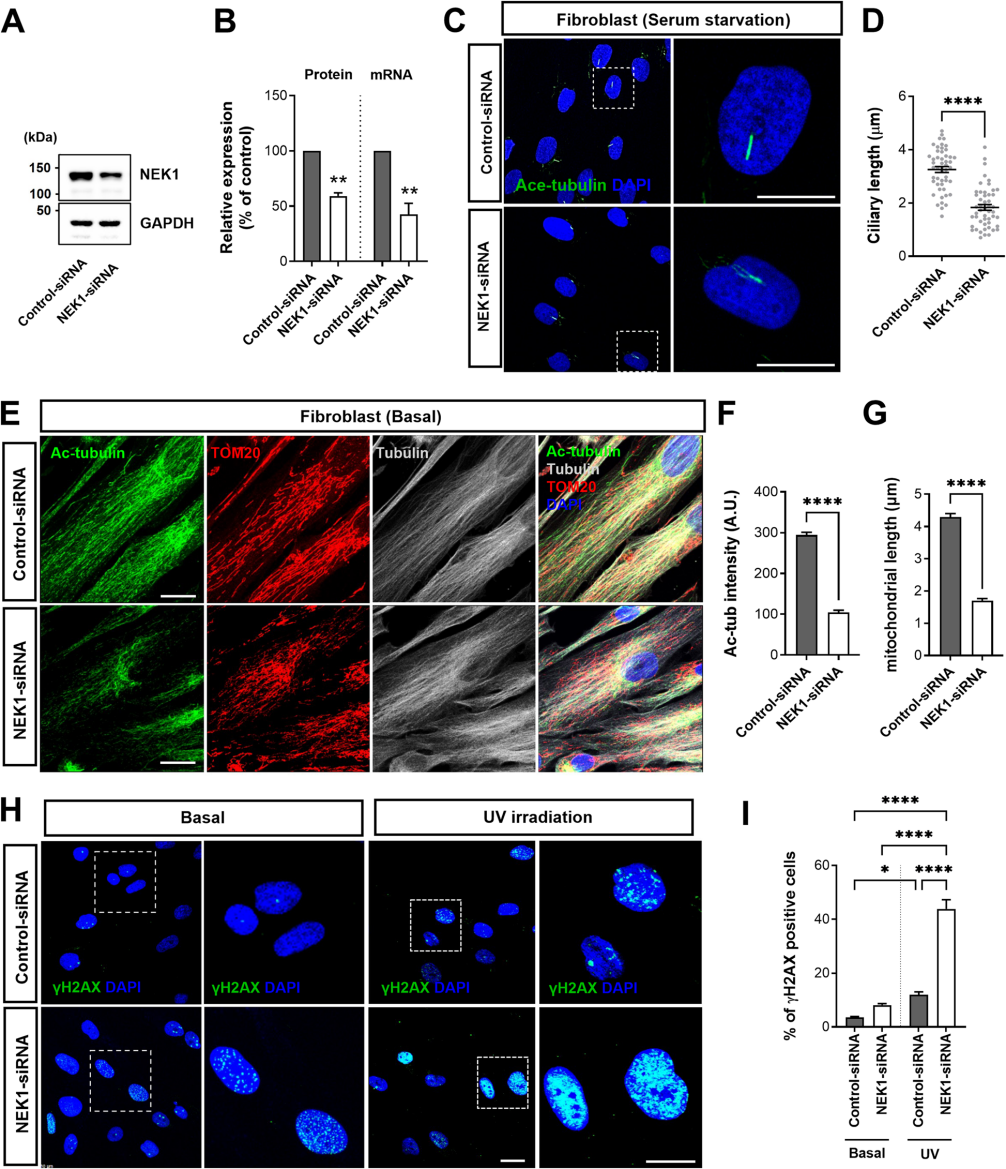
Loss of *NEK1* due to siRNA knockdown in the fibroblasts affects cilia formation, tubulin acetylation, mitochondrial distribution, and DNA damage repair. **A** Western blot analysis of NEK1 in the *NEK1* knockdown (KD) lysates using a siRNA in the control fibroblasts. GAPDH was used as a loading control. **B** Quantification of normalized *NEK1* protein and mRNA expression. Data represent mean ± SEM (from three independent experiments). Comparisons were made against the control-siRNA (**P < 0.01; Student’s *t-*test). **C** Representative fluorescence images of the primary ciliary formation by *NEK1* KD in the control fibroblasts were stained with acetylated α-tubulin (green, ciliary axoneme marker) and DAPI (blue). The right panels illustrate higher magnification views of the white box regions. Scale bar: 10 µm. **D** Quantification of the ciliary length in C. The >100 cells per condition were quantified per replicate experiment (*n* = 3). Data represent mean ± SEM. Comparisons were made against the control-siRNA (****P < 0.0001; Student’s *t-*test). **E** Representative fluorescence images of the tubulin acetylation and mitochondrial distribution by *NEK1* KD in the control fibroblasts. Cells were stained with acetylated α-tubulin (red), TOM20 (green, mitochondrial marker), α-tubulin (gray), and DAPI (blue). The lower panels illustrate the higher magnification views of the white box regions. Scale bar: 10 µm. **F** Quantification of acetylated α-tubulin (Ac-tub) intensity (**F**) and the mitochondrial length (**G**) described in E. The >50 cells per condition were quantified per replicate experiment (*n* = 3). Data represent mean ± SEM. Comparisons were made against the control-siRNA (****P < 0.0001; Student’s *t-*test). **H** Representative fluorescence images of DNA damage response in the *NEK1* KD fibroblasts. Cells were fixed either in basal condition (without irradiation) or at 24 h after UV irradiation. Cells were stained with γH2AX (S139) (green, DNA damage marker) and DAPI (blue). The right panels show the higher magnification views of the white box regions. Scale bar: 10 µm. **I** Quantification of the γH2AX-positive cells in G. The >50 cells per condition were quantified per replicate experiment (*n* = 3). Data represent mean ± SEM. Comparisons were made against the control-siRNA (*P < 0.05, ****P < 0.0001; one-way ANOVA with post-hoc Tukey’s test)

Our study shows that functional defects caused by ALS-linked *LOF* or splicing variants of *NEK1* induce alterations in primary cilia formation, tubulin acetylation, mitochondrial dynamics, and DNA damage repair. We further investigated LOF effects on *NEK1* knockdown (KD) using siRNA in the control fibroblasts (Fig. 7). *NEK1* mRNA expression and protein levels were reduced by 50% in the *NEK1* KD fibroblast (Fig. 7A and B). Under serum starvation, *NEK1* KD cells exhibited abnormal primary ciliary morphology with shortened cilia (Fig. 7C and D). Additionally, we observed a reduction in tubulin acetylation and abnormal mitochondrial morphology in the *NEK1* KD cells (Fig. 7E-G). Finally, the *NEK1* KD cells exhibited increased γH2 AX phosphorylation (S139) upon UV irradiation (Fig. 7H and I). Subsequently, we confirmed these results using *NEK1* KD in the SH-SY5Y neuronal cells (Fig. S6A–C). Furthermore, we examined the expression of the regulatory proteins of the G1-to S-phase cell cycle under starvation conditions after *NEK1* KD in the SH-SY5Y cells to verify the regulation of the cell cycle exit programs observed in quiescent neuronal cells. Interestingly, *NEK1* KD resulted in the hyperactivation of G1-to-S phase cell cycle regulatory proteins, including CDK4, cyclin D1, and RB phosphorylation, compared to the control cells (Fig. S6D and E). We propose that quiescent cells induce cell cycle re-entry as a consequence of NEK1 loss. Additionally, we evaluated the potential for NEK1 expression to ameliorate the defects observed in fibroblasts with the *NEK1*-LOF variant (p.E853Rfs*9). Overexpression of human NEK1 partially but significantly rescued abnormal cilia assembly, tubulin acetylation, abnormal mitochondrial distribution, and DNA damage repair in *NEK1*-*LOF* patient fibroblasts (Fig. S7). These data suggest that the ALS-associated *NEK1*-LOF variant results in *NEK1* haploinsufficiency.

### HDAC6 inhibition rescues NEK1-dependent deficits in patient fibroblasts carrying the *NEK1*-LOF variants

**Fig. 8:**
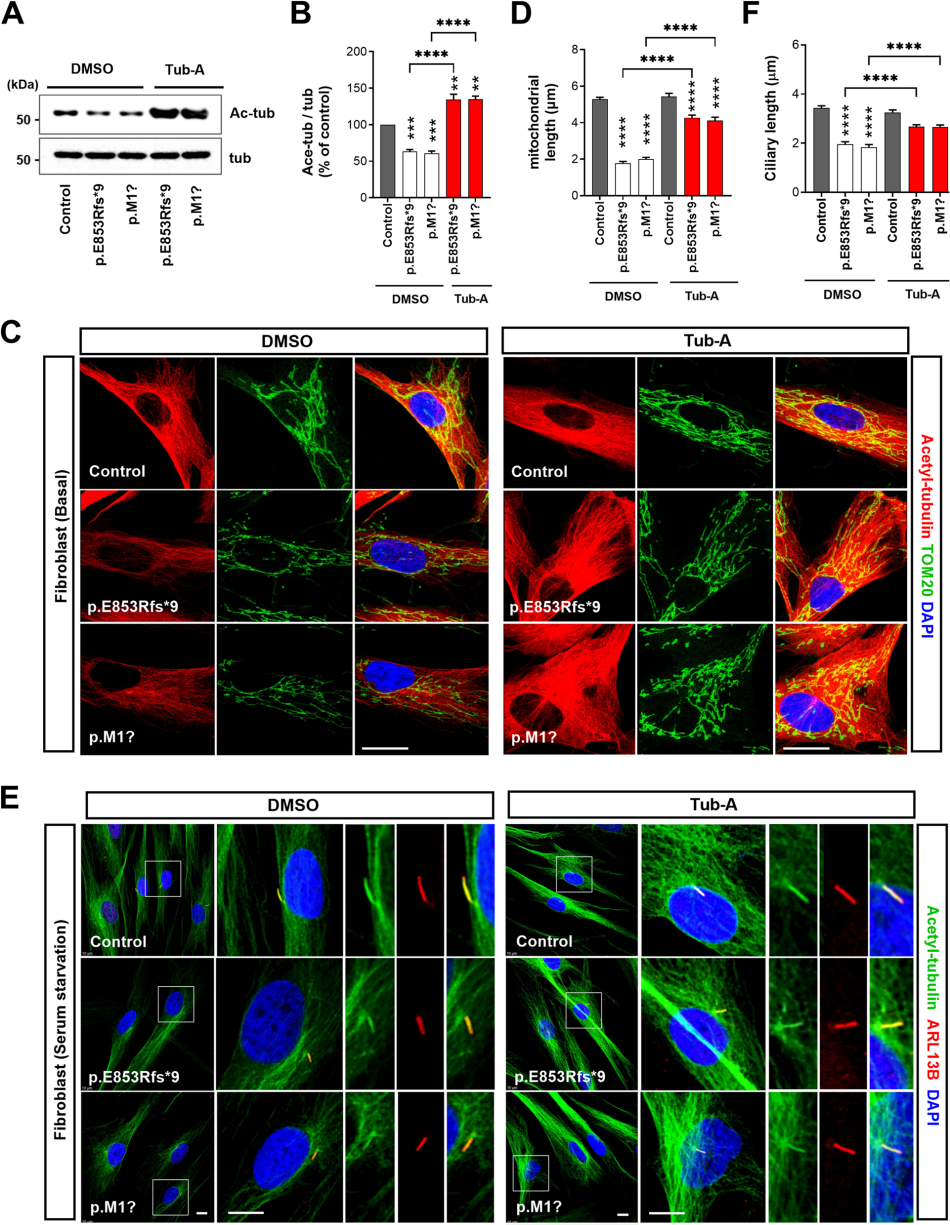
*NEK1*-LOF abnormality in the ALS fibroblasts rescued by pharmacological inhibition of HDAC6. **A** Western blot analysis of the lysates from DMSO or tubastatin A (Tub-A)-treated control and *NEK1*-LOF (p.E853Rfs*9 and p.M1?) patient fibroblasts using anti-acetylated α-tubulin (Ac-tub) and anti-α-tubulin (tub) antibodies. α-tubulin (tub) was used as a loading control. **B** Quantification of the normalized acetylated α-tubulin (Ac-tub) protein expression. Ac-tub intensities were normalized to that of the α-tubulin (tub). Data represent mean ± SEM (from three independent experiments). Comparisons were made against the DMSO-treated control (**P < 0.01, ***P < 0.001, ****P < 0.0001; one-way ANOVA with post-hoc Tukey’s test). **C** Representative fluorescence images of the tubulin acetylation and mitochondrial distribution in control and *NEK1*-LOF (p.E853Rfs*9 and p.M1?) patient fibroblasts by Tub-A treatment. Cells were treated with DMSO or 1 μM Tub-A for 24 h and then stained with anti-acetylated α-tubulin antibody (red), TOM20 (green, mitochondrial marker), and DAPI (blue). Scale bar: 10 µm. **D** Quantification of the mitochondrial length in C. The >50 cells per condition were quantified per replicate experiment (*n* = 3). Data represent mean ± SEM. Comparisons were made against the DMSO-treated control (****P < 0.0001; one-way ANOVA with post-hoc Tukey’s test). **E** Representative fluorescence images of the primary cilia formation in the control and *NEK1*-LOF (p.E853Rfs*9 and p.M1?) patient-derived fibroblasts pretreated with DMSO or 1 μM Tub-A. Cells were stained with acetylated α-tubulin (green, ciliary axoneme marker) and ARL13B (green, ciliary membrane marker) after serum starvation for 48 h. The right panels show the higher-magnification views of the primary ciliary regions. Scale bar: 10 µm. **F** Quantification of the ciliary length in **E**. The >100 cells per condition were quantified per replicate experiment (*n* = 3). Data represent mean ± SEM. Comparisons were made against the DMSO-treated control (****P < 0.0001; one-way ANOVA with post-hoc Tukey’s test)

**Fig. 9:**
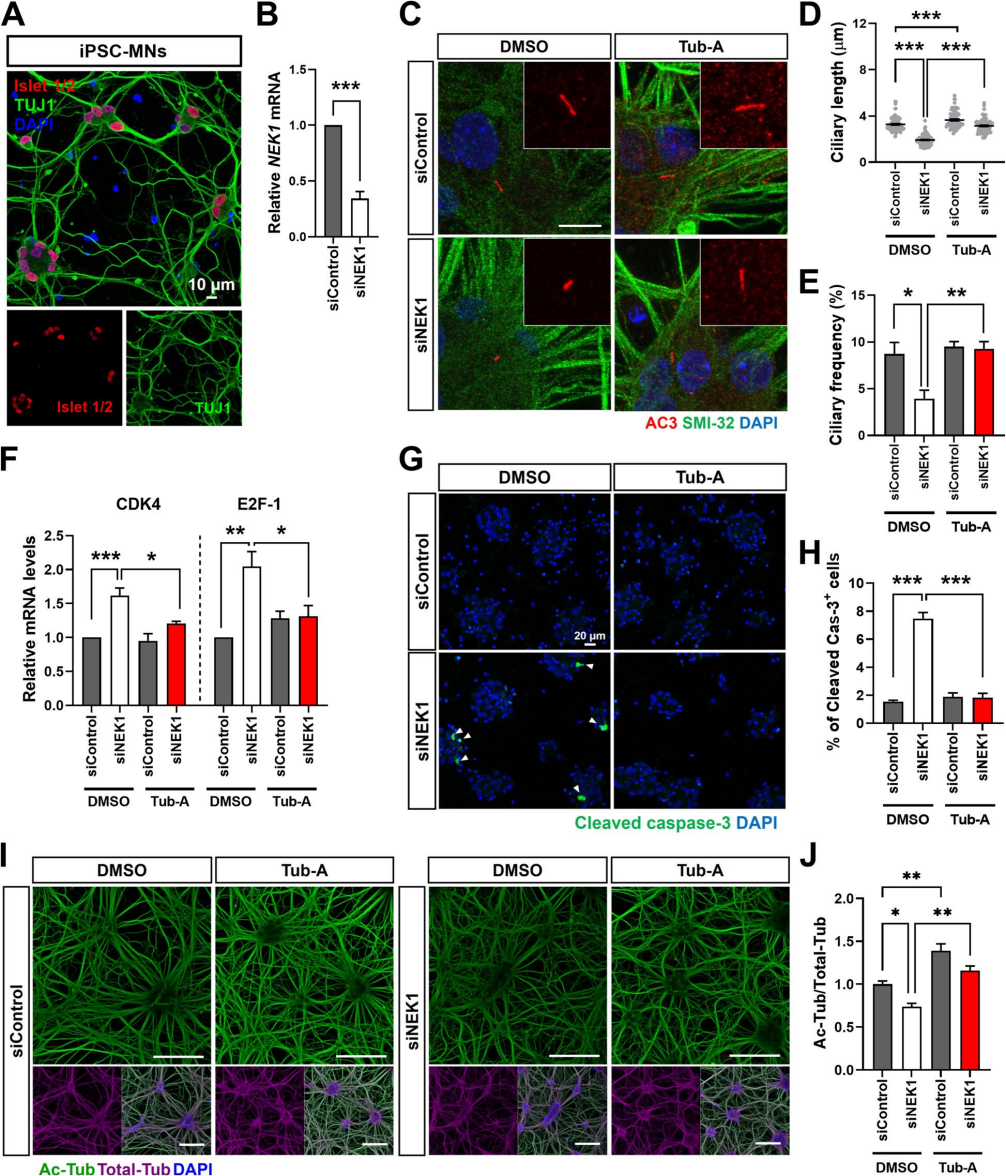
HDAC6 inhibition rescues ciliary defects, tubulin acetylation, and neuronal cell death in *NEK1*-knockdowned iPSC-MNs. **A** Representative image of iPSC-MNs stained with Islet1/2 (red), TUJ1 (green), and DAPI (blue). Scale bar: 10 µm. **B** Fold change in *NEK1* mRNA levels in control siRNA (siControl) and *NEK1* siRNA (siNEK1)-treated iPSC-MNs. Data represent mean ± SEM (*n* = 5). Comparisons were made against the siControl (***P < 0.001; Student’s *t-*test). **C** Representative fluorescence images of primary cilia formation from iPSC-MNs. Cells were treated with DMSO or 1 μM Tub-A for 24 h and then stained with anti-ACIII (red, neuronal cilia marker), SMI-32 (green, motor neuron marker), and DAPI (blue). Scale bar: 10 µm. **D**-**E** Quantification of the ciliary length (**D**) and ciliary frequency (**E**) in **C**. The >90 cells per condition were quantified per replicate experiment (*n* = 4). Data represent mean ± SEM. Comparisons were made against the DMSO-treated siControl (*P < 0.05, **P < 0.01, ****P < 0.0001; one-way ANOVA with post hoc Tukey’s tests). **F** Relative mRNA levels of the cell cycle regulators (*CDK4* and *E2F-1*) from G1 to S phase in siControl and siNEK1-treated iPSC-MNs following DMSO or Tub-A treatment. Data represent mean ± SEM (*n* = 4). Comparisons were made against the DMSO-treated siControl (*P < 0.05, **P < 0.01, ***P < 0.001; one-way ANOVA with post-hoc Tukey’s test). **G**. Representative fluorescence images stained with cleaved caspase‐3 (green) and DAPI (blue) in siControl and siNEK1-treated iPSC-MNs following DMSO or Tub-A treatment. DAPI (blue) was used to detect nuclei. Scale bar: 20 µm. **H** Quantification of cleaved Cas-3-positive cells in G. Data represent mean ± SEM (*n* = 8). Comparisons were made against the DMSO-treated siControl (***P < 0.001; one-way ANOVA with post hoc Tukey’s tests). **I** Representative fluorescence images of the acetylated α-tubulin (green) and α-tubulin (purple) in siControl and siNEK1-treated iPSC-MNs following DMSO or Tub-A treatment. DAPI (blue) was used to detect nuclei. Scale bar: 100 µm. **J** Quantification of acetylated α-tubulin (Ac-Tub) intensity in I. Data represent mean ± SEM (*n* = 3). Comparisons were made against the DMSO-treated siControl (*P < 0.05, **P < 0.01; one-way ANOVA with post hoc Tukey’s tests)

Deacetylation of axoneme microtubules by HDAC6 activation is crucial for promoting ciliary disassembly [55]. Our result demonstrated that ALS-linked *NEK1* variants upregulated HDAC6 activity under serum-starvation conditions (Fig. 3H). Therefore, we evaluated the therapeutic effects of tubastatin A (Tub-A), a selective HDAC6 inhibitor, on the functional defects observed in the *NEK1*-LOF variants. Control and patient fibroblasts were treated with 1 μM Tub-A (or DMSO), followed by western blotting to assess the acetylated α-tubulin level and immunostaining with acetylated α-tubulin and TOM20 to assess MT and mitochondrial morphology, respectively. *NEK1*-LOF variant fibroblasts treated with Tub-A considerably elevated the acetylated α-tubulin expression and rescued abnormalities in mitochondrial distribution and length (Fig. 8A–D). We pretreated control and patient fibroblasts with Tub-A (or DMSO) under serum starvation conditions and stained with acetylated α-tubulin and ARL13B. Compared with the DMSO-treated control, the Tub-A treatment restored most of the shortening caused by impaired ciliogenesis in the *NEK1*-LOF fibroblasts (Fig. 8E and F). In addition, the Tub-A or BAPTA treatment partially rescued the impaired DNA damage repair after UV irradiation in patient fibroblasts of ALS-linked *NEK1* variants (Fig. S8A and B). Finally, to validate the effects of *NEK1* loss in disease-relevant cell models, we assessed whether the primary cilia and microtubule-related phenotypes obtained from patients fibroblasts are reproducible in human iPSC-MNs using previously established protocols [41, 42]. We confirmed that iPSC-MNs expressed neuronal (TUJ1) and motoneuronal (ISL-1, SMI-32) markers (Fig. 9A and C). To investigate the effect of reduced levels of *NEK1*, we performed knock-down (KD) experiments using siRNA targeting *NEK1* and scrambled control (Fig. 9B). We found that *NEK1* KD MNs exhibit shortened ciliary morphology, decreased tubulin acetylation, altered cell-cycle progression, and apoptotic cell death (Fig. 9C-J). Furthermore, these defects were restored by Tub-A treatment (Fig. 9C-J). These data from iPSC-MNs combined with patient fibroblasts data suggest that ALS-linked *NEK1* LOF mutations may contribute to disease pathogenesis by affecting primary ciliogenesis, tubulin acetylation, and neuronal survival, and that HDAC6 inhibition has indeed a beneficial effect on the neuronal survival with *NEK1* mutations.

## Discussion

NEK1 is a multifunctional kinase involved in diverse cellular processes, including primary cilia formation, cell cycle regulation, DDR, and microtubule dynamics. Genetic studies have shown that *NEK1* LOF variants can lead to haploinsufficiency and contribute to ALS development [3, 4]. In this paper, we analyzed the whole-exome sequences of 920 Korean patients with sporadic ALS and identified 16 *NEK1* variants in 23 patients (23/920, 2.5%). Concerning phenotypical aspects of patients with *NEK1* variants, clinical manifestations including age of onset, severity, and disease progression compared to sporadic ALS cases were not significantly different but only survival times were shorter in patients with *NEK1* LOF variants (Fig. S9). Functional studies have linked *NEK1* mutations to disruptions in DDR, microtubule homeostasis, and nucleocytoplasmic transport in iPSC-MN models [36, 37], but have not yet provided sufficient information to understand MN degeneration. Herein, considering the NEK1’s diverse cellular functions, we provided evidence that ALS-linked *NEK1* mutations cause primary cilia- and microtubule-related defects. First, we demonstrated that ALS-linked *NEK1* variants induce abnormal structure and function of primary cilia by activating the ciliary disassembly axis Ca^2+^-AurA-HDAC6, which in turn alters cell cycle progression. Second, *NEK1* variants reduced tubulin acetylation, caused mitochondrial dysfunction, and impaired the DDR, all of which are closely linked to ciliary defects [68]. Furthermore, HDAC6 inhibitor treatment restored defective primary cilia, tubulin acetylation, and abnormal mitochondrial distribution in *NEK1* LOF patient-derived fibroblasts and iPSC-MNs model, suggesting its potential as a therapeutic target for ALS.

Primary cilia, characterized by their non-motile nature, play a pivotal role in the development of multicellular tissues and organs. This importance is highlighted by the severe consequences of ciliopathies, which result from mutations in molecules associated with cilia [26]. However, primary cilia’s function in neurons that are differentiated, non-dividing, and post-mitotic is not well understood. Primary cilia have recently been shown to be involved in processes such as mitochondrial dynamics and the DDR, which are crucial for maintaining neuronal homeostasis [68]. Primary cilia are continuously maintained on the cell surface in neurons to act as signaling antennae for various external stimuli and prevent cell cycle re-entry, thereby maintaining neuronal identity and homeostasis [18]. Furthermore, abnormalities in primary cilia and ciliary signaling pathways in neurons play important roles in aging and age-related neurodegenerative diseases [27, 28]. The proportion of ciliated MNs was previously shown to substantially decrease in the primary culture and the lumbar spinal cord of the SOD1-G93A ALS mouse model [29]. Additionally, SOD1-G93A overexpression decreased signaling in the Shh pathway, making SOD1-G93A cells more vulnerable to oxidative stress. Treating SOD1-G93A cells with Shh or Shh agonists offers cytoprotective benefits [30]. These findings suggest that primary ciliary dysfunction and ALS development appear to be closely related. To date, the identification of *NEK1*, a gene known to play a key role in ciliogenesis and cell cycle regulation, as an ALS risk gene suggests that disruptions in these cellular processes contribute to ALS pathogenesis. However, whether they are regulated by ALS mutations in this gene is not well understood. Our data clearly show that patient fibroblasts carrying ALS-linked *NEK1* variants, two LOF variants (p.E853Rfs*9 and p.M1?) and one synonymous splicing variant (p.Q132=) caused ciliary morphology shortening and Shh signaling impairment. Ciliary disassembly regulation is widely known to be driven by Aura’s kinase activity, which is stimulated by Ca^2+^/Calmodulin [55, 56]. This mechanism controls HDAC6 activity. Our results are consistent with the observation that fibroblasts from patients carrying ALS-linked *NEK1* variants cause ciliary disassembly by activating AurA phosphorylation and HDAC6 through increased cytoplasmic calcium levels. MNs vulnerable to ALS are well known to be highly sensitive to increased intracellular calcium levels, which occur due to the loss of calcium-buffering proteins [57]. This, combined with our results, suggests that these neurons are also sensitive to ciliary homeostasis maintenance. A recent study described that reduction or ALS-linked mutation of *C21orf2* leads ciliary dysfunction in MNs [69]. C21orf2 is localized at the basal body of the primary cilia, with ALS mutations altering this localization. Reduced *C21orf2* levels cause shorter primary cilia and impair Shh signaling, similar to the defects seen with *NEK1* mutations. Furthermore, C21orf2 overexpression rescued both ciliary defects and neuromuscular junction formation, suggesting primary cilia dysfunction contributes to motor neuron degeneration in ALS. Another group reported that iPSC-MNs from C21orf2-V58L-ALS patients exhibited elevated apoptosis, dysregulated DNA damage responses, mitochondrial dysfunction, and altered neuronal excitability [70]. Notably, C21orf2-V58L induced downregulation of NEK1, further implicating NEK1 in ALS pathology. These studies suggest that the pathogenic effects of *C21orf2* mutations may be linked to NEK1 loss and associated ciliary dysfunction.

Given the connectivity between the primary cilia and cell cycle processes, defective cell cycle regulation has also been observed in several neurodegenerative diseases in which chronic neuronal cell loss is a characteristic feature [71, 72]. Neurons, unlike many cell types, remain in a resting state in the adult nervous system and are considered to permanently lose their ability to divide and proliferate once completely differentiated. Nevertheless, several genes associated with regulating the G1/S transition, such as cyclin D1, Cdk4, Rb, and E2Fs, are found in the normal adult brain [71, 72]. These genes may be activated in response to pathological changes, including DNA damage, oxidative stress, and excitotoxicity, triggering aberrant cell cycle re-entry and apoptosis in specific neurons. These processes involve increased cyclin D-Cdk4/6 activity and deregulation of the E2F transcription factor, resulting in cell death. Our data clearly demonstrated an abnormal increase in these cell cycle regulators in ALS patient fibroblasts exhibiting ciliary defects. This finding suggests that ALS-linked *NEK1* variants cause ciliary abnormalities and aberrant cell cycle entry, leading to motor neuron degeneration. However, whether NEK1 regulates ciliary dynamics by controlling the cell cycle or whether it controls cell cycle progression by regulating ciliary dynamics remains to be elucidated. Further research is needed to clarify this issue.

Ciliogenesis is tightly regulated by the changes in the post-translational modifications of α-tubulin because primary cilia are dynamic microtubule-based organelles [73]. Additionally, the impairment of microtubule dynamics is a common mechanism in neurodegenerative diseases as it plays an important role in mitochondrial transport [74]. *NEK1* variants associated with ALS resulted in the impairment of microtubule dynamics through a decrease in acetylated α-tubulin and subsequent mitochondrial abnormalities. HDAC6, a class II histone deacetylase, plays a pivotal role in regulating cytoskeletal dynamics by deacetylating α-tubulin and cortactin [75]. The significance of HDAC6 in ALS was demonstrated through HDAC6 genetic deletion, which substantially slowed disease progression and extended survival in the SOD1-G93A mouse model [76]. Furthermore, defects caused by FUS or TDP-43 mutations have recently suggested to be regulated by HDAC6 inhibition [77, 78], indicating that HDAC6 may be a valuable target for ALS treatment. HDAC6 is localized in the cytoplasm and basal body and has been identified as an important driver of ciliary disassembly [79]. HDAC6 plays a critical role in deacetylating α-tubulin to regulate axoneme microtubule dynamics in the primary cilia [80]. As previously mentioned, patient fibroblasts carrying ALS-linked *NEK1* variants demonstrated increased HDAC6 activity compared to the control fibroblasts. Thus, we hypothesized that HDAC6 inhibition may be a promising therapeutic approach for treating ALS with *NEK1* mutations. Herein, we discovered that HDAC6 inhibition by tubastatin A treatment restored ciliary length, the proportion of ciliated cells, and mitochondrial defects by increasing α-tubulin acetylation. Our study has several limitations. One notable limitation is the relatively low baseline proportion of primary cilia observed in our iPSC-MNs. Recent systematic analysis has demonstrated that the frequency of primary cilia in iPSC-derived neuronal cultures can vary substantially depending on the differentiation protocol, culture conditions, and maturation stage [81]. In our study, although the ciliation rate in iPSC-MNs was lower compared to fibroblasts, a similar trend was observed where *NEK1* knockdown (KD) led to a reduction in both ciliary length and ciliary frequency, as compared to control neurons. Therefore, the relatively low ciliation rate observed in our system is likely attributable, at least in part, to technical variables associated with the differentiation protocol and culture conditions, rather than reflecting an inherent deficiency in ciliogenesis. Future studies optimizing motor neuronal maturation protocols and environmental conditions will be important to further improve the ciliary frequency and more faithfully model ciliary pathology in ALS. Another limitation of our study is that we have not yet directly demonstrated the relationship between calcium homeostasis and ciliary defects in MNs. We hypothesize that *NEK1* mutations drive calcium-dependent ciliary disassembly in MNs, supported by two aspects. First, previous studies have highlighted the diverse post-mitotic functions of AurA in neurons, beyond its well-established role in cell division [82, 83]. These studies show that AurA is not only expressed in neurons but also plays critical roles in microtubule organization, neuronal migration, and synaptic plasticity. While the role of AurA in neurons has been less extensively studied compared to calcium ions and HDAC6, the calcium-AurA-HDAC6 signaling pathway identified in fibroblasts suggests that a similar mechanism could operate in neurons. Second, calcium homeostasis is crucial for motor neuron physiology, and dysregulation of calcium signaling is known to contribute to selective motor neuron vulnerability in ALS. In proliferating cells, increased cytosolic calcium binds to calmodulin, which activates AurA. AurA then phosphorylates and activates HDAC6, promoting ciliary disassembly by deacetylating axonemal α-tubulin. Based on these findings, we hypothesize that the calcium-AurA-HDAC6 pathway, which regulates ciliary dynamics in fibroblasts, may similarly regulate ciliogenesis in MNs. Abnormal calcium regulation in MNs could lead to ciliary defects via this pathway, contributing to ALS pathogenesis. However, the precise role of this pathway in MNs and its impact on ciliary function in ALS remain to be fully elucidated. Our findings in fibroblasts demonstrate that *NEK1* mutations activate the calcium-AurA-HDAC6 pathway, leading to abnormal ciliogenesis, but further research is required to validate this pathway in MNs and explore its relevance to ALS pathology. Finally, while we have observed structural and functional mitochondrial phenotypes in patient fibroblasts, the direct connection between ciliary dysfunction and mitochondrial abnormalities remains to be fully elucidated. Emerging evidence suggests a complex interplay between primary cilia and mitochondria. Several studies have demonstrated that mitochondrial dysfunction can alter ciliary gene expression, particularly in brain cell types. For instance, viral-induced downregulation of the mitochondrial fusion gene *mitofusin 2* (MFN2), a key protein involved in mitochondrial fusion and mitochondria–endoplasmic reticulum interactions, in dopamine D1 neurons was shown to modify ciliary gene expression and cilium structure [84]. Similarly, mitochondrial DNA depletion in astrocytes significantly altered the structure of the primary cilium [85]. These findings provide evidence for a functional interaction between mitochondria and cilia, highlighting the role of mitochondrial dynamics in regulating ciliary structure in the brain. Conversely, primary cilia and ciliary signaling pathways also appear to regulate mitochondrial function. In thyroid cancer cell lines, deletion or knockdown of *IFT88* and *KIF3A* impaired ciliogenesis, enhanced VDAC1 oligomerization following VDAC1 overexpression, and disrupted mitochondrial morphology and function, ultimately promoting mitochondria-mediated apoptosis [86]. Consistent with these observations, our data show that mitochondrial dynamics and gene expression related to ciliogenesis are altered in patient fibroblasts carrying *NEK1* mutations. Although we have not yet directly demonstrated the link between calcium dysregulation, ciliary defects, and mitochondrial dysfunction in iPSC-MNs, the available literature, together with our observations in fibroblasts, support the hypothesis that these processes are interconnected. Future studies will be necessary to further investigate the role of NEK1 in mitochondrial function and ciliary dynamics in MNs.

Overall, our study represents an initial exploration of the involvement of defective primary ciliary homeostasis in ALS pathogenesis and underscores the therapeutic potential of HDAC6 inhibition for ALS.

## Supplementary Information

Supplementary Material 1.

## Data Availability

The IRB of Hanyang University Hospital will review all requests for raw and analyzed data and related materials to determine whether each request is subject to any intellectual property or confidentiality restrictions. Data supporting the results of this study can be obtained from the corresponding author upon request.
