# Supplementary material for: Mutations in NEK1 cause ciliary dysfunction as a novel pathogenic mechanism in amyotrophic lateral sclerosis
Source: Mol Neurodegener. 2025 May 20;20:59. doi: 10.1186/s13024-025-00848-7 (PMC12090460; doi:10.1186/s13024-025-00848-7)
Supplement: Supplementary file 1 — Supplementary Material 1. [file 13024_2025_848_MOESM1_ESM.docx]

Supplementary Information

Title

**Mutations in *NEK1* cause ciliary dysfunction as a novel pathogenic mechanism in amyotrophic lateral sclerosis**

Min-Young Noh^1, †^, Seong-il Oh^2, †^, Young-Eun Kim^3, †^, Sun Joo Cha^4^, Wonjae Sung^1^, Ki-Wook Oh^1^, Yurim Park^5,6^, Ji Young Mun^5^, Chang-Seok Ki^7^, Minyeop Nahm^4*^ and Seung Hyun Kim^1,8*^

Emails of the corresponding and the co-corresponding authors: [kimsh1@hanyang.ac.kr](mailto:kimsh1@hanyang.ac.kr) (Seung Hyun Kim); [nmy92@kbri.re.kr](mailto:nmy92@kbri.re.kr) (Minyeop Nahm)

**^†^These authors contributed equally to this work.**


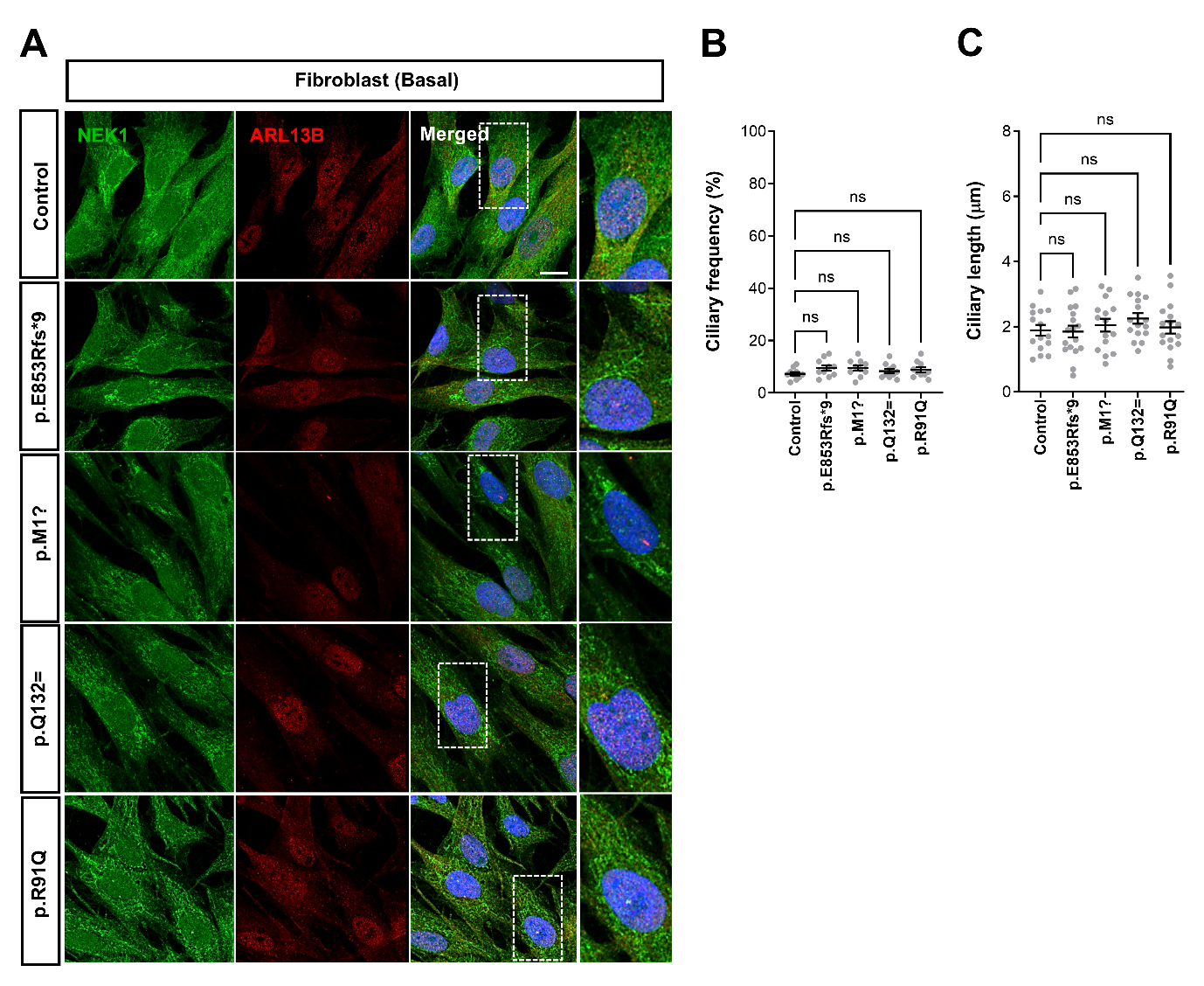


**Fig. S1.** **NEK1 expression and primary ciliogenesis in control and patient fibroblasts under basal condition.**

**A.** Representative fluorescence images of endogenous NEK1 (green) and ARL13B (red, cilia marker) in the control and patient fibroblasts under basal culture conditions. The right panels illustrate higher magnification views of white box regions. Nuclei were stained with DAPI. Scale bar: 10 µm. **B - C.** Quantification of the ciliary frequency (**B**) and the ciliary length (**C**) in A. The >100 cells per condition were quantified per replicate experiment (n = 3). Data represent mean ± SEM. Comparisons were made against the control (ns: not significant; one-way ANOVA with post-hoc Tukey’s tests).


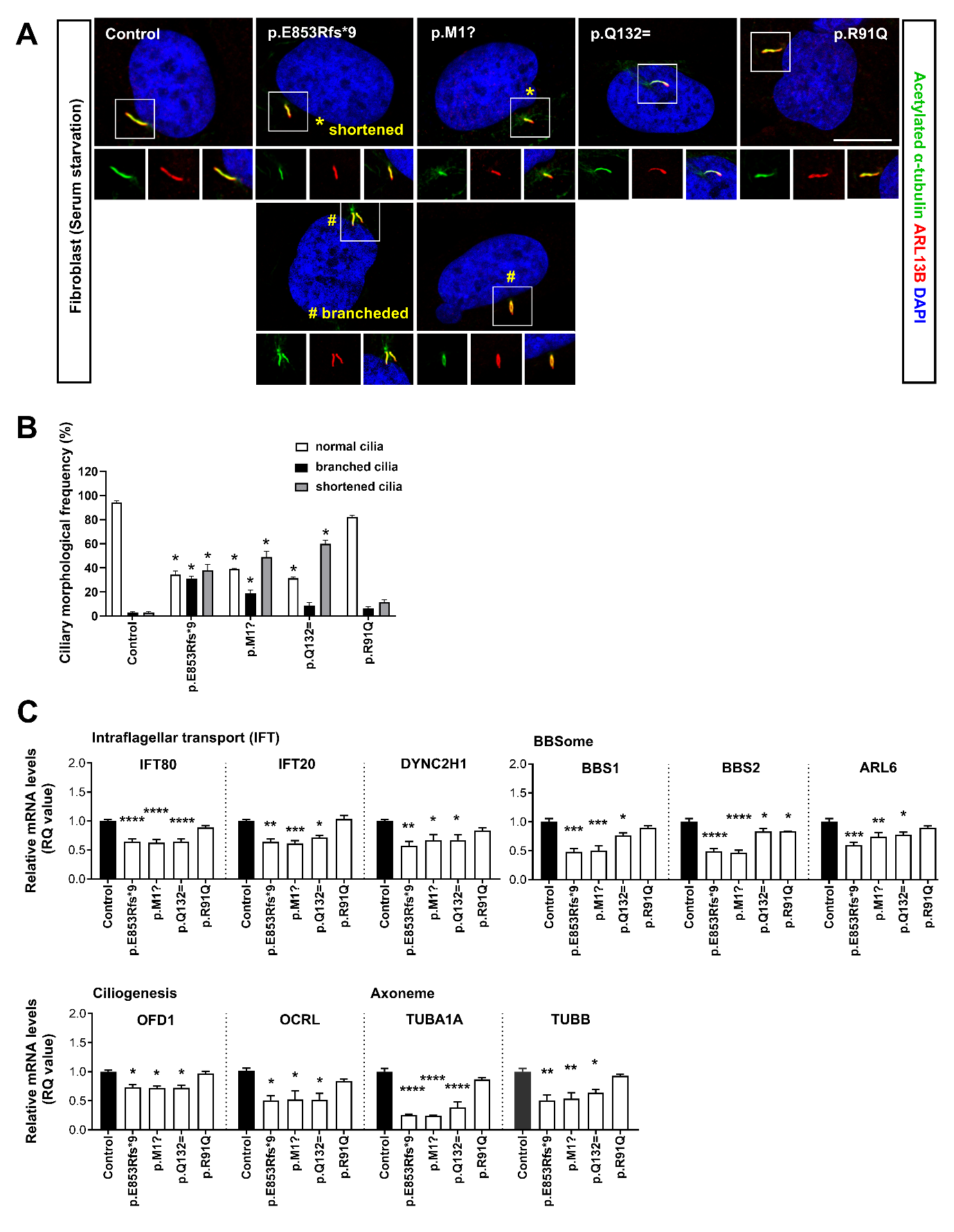


**Fig. S2.** **ALS-linked *NEK1* variants present primary cilia dysfunction in patient fibroblasts.**

**A**. Representative fluorescence images of the primary ciliary formation in the control and patient fibroblasts stimulated with serum starvation for 48 h. Cells were stained with acetylated α-tubulin (red, ciliary axoneme marker), ARL13B (green, cilia marker), and DAPI (blue). The bottom panels indicate higher magnification views of the primary ciliary regions. The abnormal ciliary morphology in patient fibroblasts carrying *NEK1*-LOF variants (p.E853Rfs*9 or p.M1?) is indicated by shortened cilia (yellow asterisks) or branched cilia (yellow sharp). Scale bar: 10 µm. **B**. Quantification of the ciliary morphological frequency in A. The >100 cells per condition were quantified per replicate experiment (n = 3). Data represent mean ± SEM. Comparisons were made against the control (**P* < 0.05; one-way ANOVA with post-hoc Tukey’s tests). **C**. Normalized gene expression levels of cilia-associated genes, including intraflagellar transport (*IFT, IFT80, IFT20, DYNC2H1*), BBSome (*BBS1, BBS2, ARL6*), ciliogenesis (*OFD1, OCRL*), and axoneme (*TUBA1A, TUBB*). mRNA levels were analyzed by qPCR in control and patient fibroblasts stimulated with serum starvation for 48 h. Data represent mean ± SEM (from three independent experiments). Comparisons were made against the control (**P* < 0.05, ***P* < 0.01, *****P* < 0.0001; one-way ANOVA with post-hoc Tukey’s test).


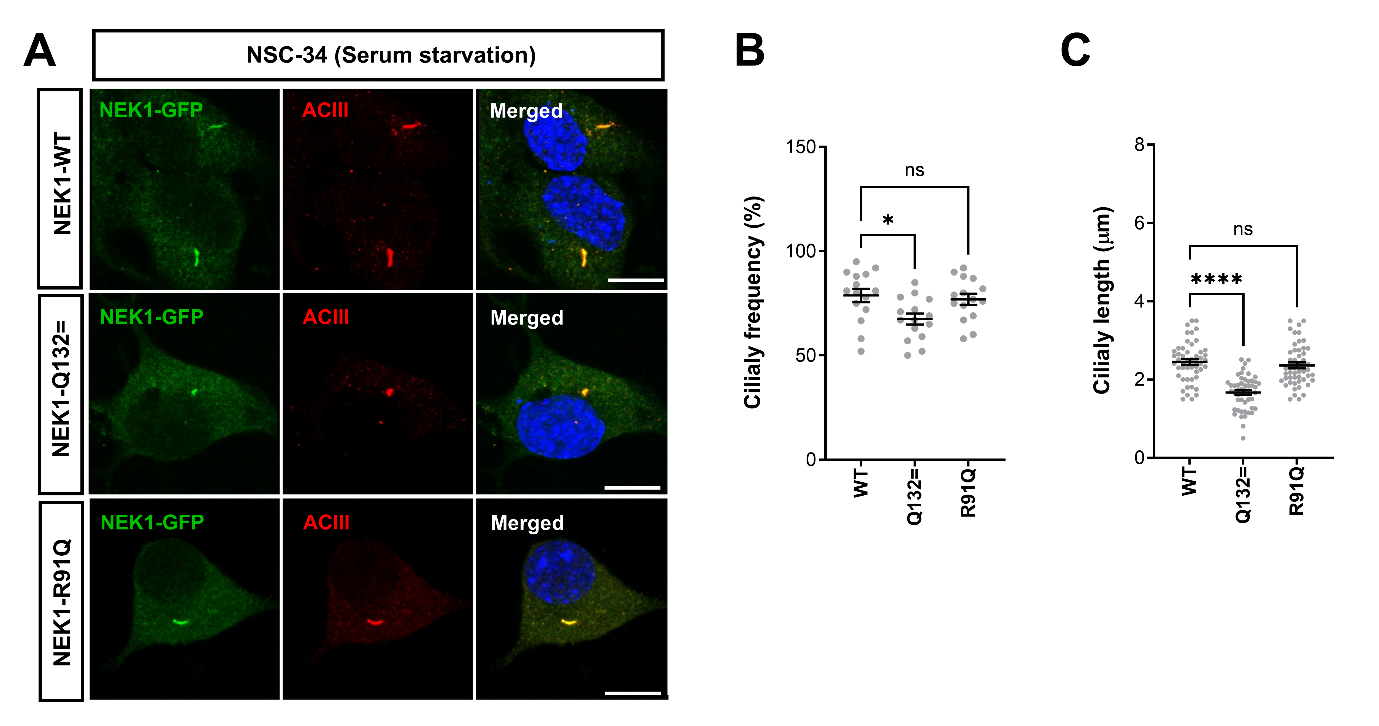


**Fig. S3.** **ALS-linked *NEK1* variants affect primary cilia assembly in motor neuronal cells.**

**A.** Representative fluorescence images of primary ciliary formation stimulated with serum starvation for 48 h after transfection of GFP-tagged NEK1 WT or its mutant forms in NSC-34 cells. Cells were stained with ACIII (red, neuronal cilia marker), GFP (green), and DAPI. Scale bar: 10µm. **B - C.** Quantification of the ciliary frequency (**B**) and the ciliary length (**C**) in A. The 50 transfected cells per condition were quantified per replicate experiment (n = 3). Data represent mean ± SEM. Comparisons were made against the NEK1-WT (ns: not significant, **P* < 0.05, *****P* < 0.0001; one-way ANOVA with post hoc Tukey’s tests).


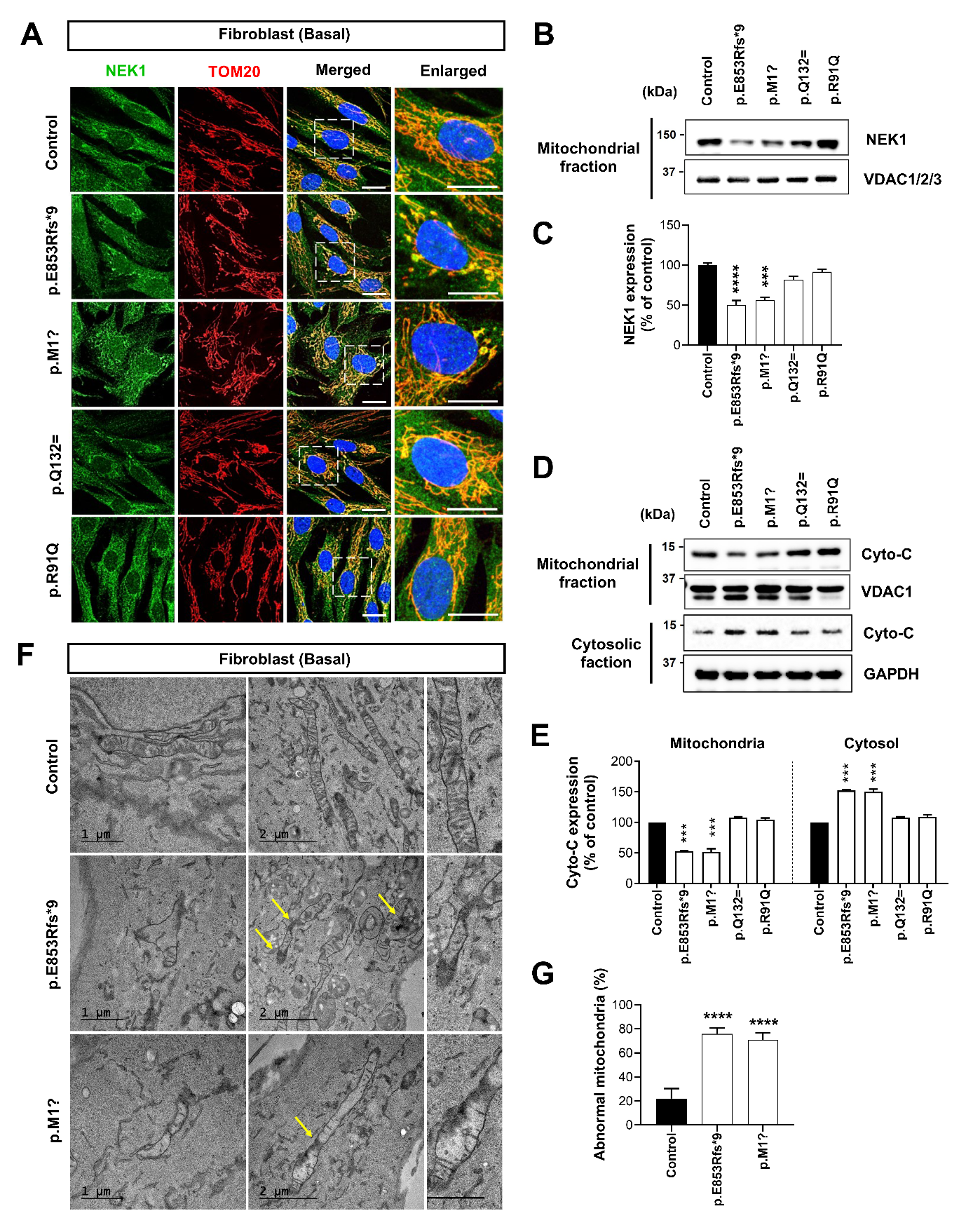


**Fig. S4.** ***NEK1-*LOF variants affect mitochondrial morphology and function in patient fibroblasts.**

**A.** Confocal analysis shows partial colocalization of endogenous NEK1 (green) and TOM20 (red, mitochondrial marker) in control and patient fibroblasts. The right panels show higher magnification views of white box regions. Scale bar: 10 µm. **B**. Western blot analysis of mitochondrial fractions from control and *NEK1* patient fibroblasts using anti-NEK1 antibody. Voltage-dependent anion channel (VDAC 1/2/3) was used as a loading control for the mitochondrial fraction. **C**. Quantification of normalized NEK1 protein expression from three independent experiments. Data represent mean ± SEM. Comparisons were made against the control (****P* < 0.001, *****P* < 0.0001; one-way ANOVA with post hoc Tukey’s tests). **D**. Western blot analysis of mitochondrial and cytosolic fractions from control and *NEK1* patient fibroblasts using anti-cytochrome C (Cyto-C) antibodies. GAPDH was used as a loading control for the cytosolic fraction, and the voltage-dependent anion channel (VDAC) was used for the mitochondrial fraction. **E**. Quantification of normalized expression of Cyto-C protein from three independent experiments. The band intensities of Cyto-C in each fraction were normalized to the mitochondrial or cytosolic Cyto-C intensities. Data represent mean ± SEM. Comparisons were made against the control (****P* < 0.001; one-way ANOVA with post hoc Tukey’s tests). **F**. Representative transmission electron microscopy (TEM) images in control and *NEK1*-LOF (p.E853Rfs*9 and p.M1?) patient fibroblasts showing mitochondrial ultrastructural defects (without cristae structures, rupture of the outer mitochondrial membrane; yellow asterisks). Mitochondrial images were shown in magnified right. Scale bar, 1 μm. **G**. Quantification of cells with abnormal mitochondrial structure from three independent experiments. Data represent mean ± SEM. Comparisons were made against the control (*****P* < 0.0001; one-way ANOVA with post hoc Tukey’s tests).


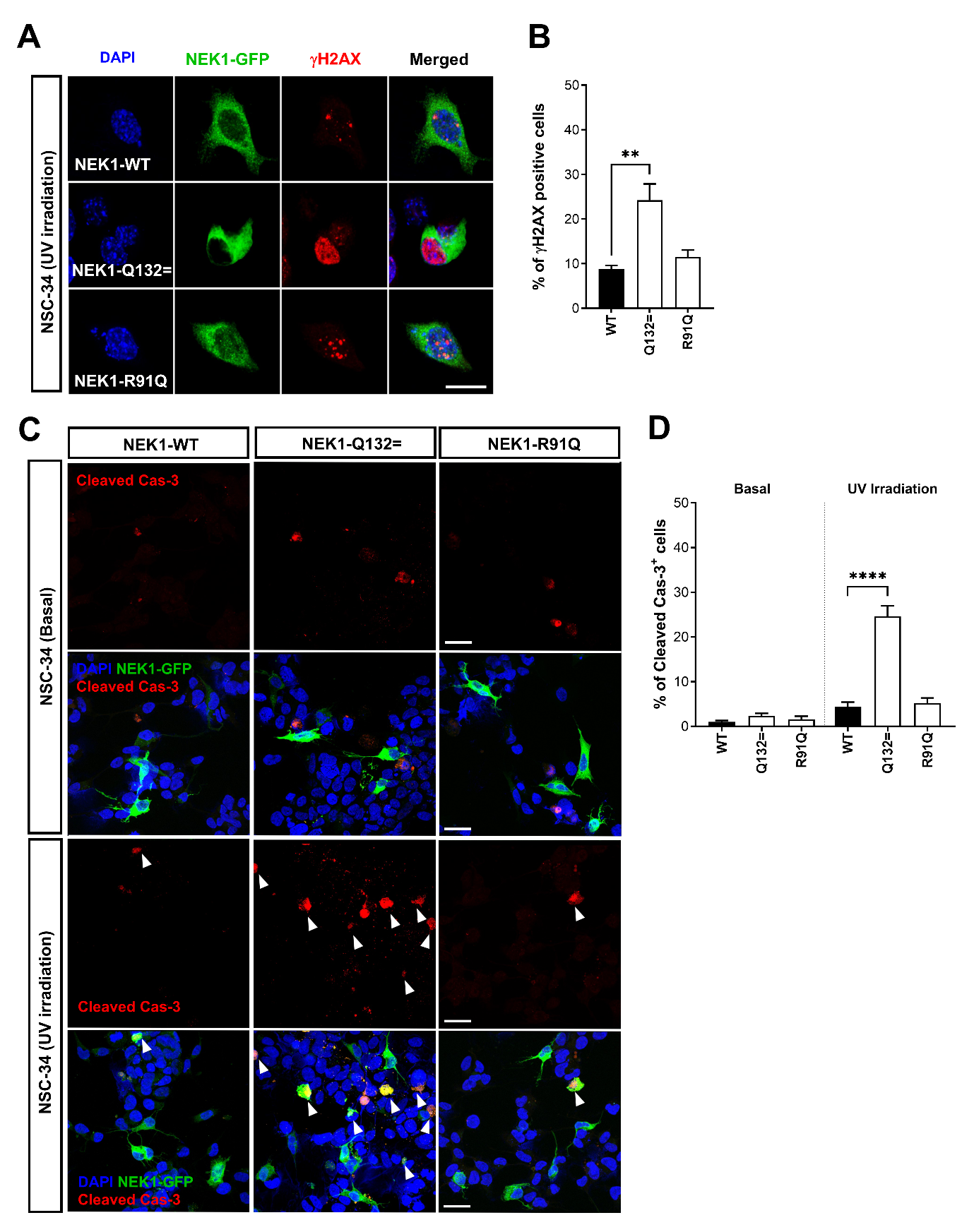


**Fig. S5.** **Expression of ALS-linked *NEK1* variants impairs DNA damage repair and induces motor neuronal cell apoptosis.**

**A.** Representative fluorescence images of NSC-34 cells transiently expressing GFP-tagged NEK1-WT or NEK1 variants (Q132= and R91Q) 24 h after 20 J/m^2^ UV irradiation. Transfected cells were stained with γH2AX (S139) (red, DNA damage marker) and DAPI (blue). Scale bar: 20 µm. **B**. Quantification of γH2AX-positive cells in A. The >50 transfected cells per condition were quantified per replicate experiment (n = 3). Data represent mean ± SEM. Comparisons were made against the WT (***P* < 0.01; one-way ANOVA with post hoc Tukey’s tests). **C**. Representative fluorescence images stained with cleaved caspase-3 (red) in NSC-34 cells transiently expressing GFP-tagged NEK1-WT or NEK1 variants (Q132= and R91Q) under basal condition (without UV irradiation) or after UV irradiation. DAPI (blue) was used to detect nuclei. Scale bar: 100 µm. **D**. Quantification of cleaved Cas-3-positive cells in C. Average cell death rates were measured as the percentage of cleaved Cas-3-positive cells in GFP-positive cells. The >50 transfected cells per condition were quantified per replicate experiment (n = 3). Data represent mean ± SEM. Comparisons were made against the control (*****P* < 0.0001; one-way ANOVA with post hoc Tukey’s tests).


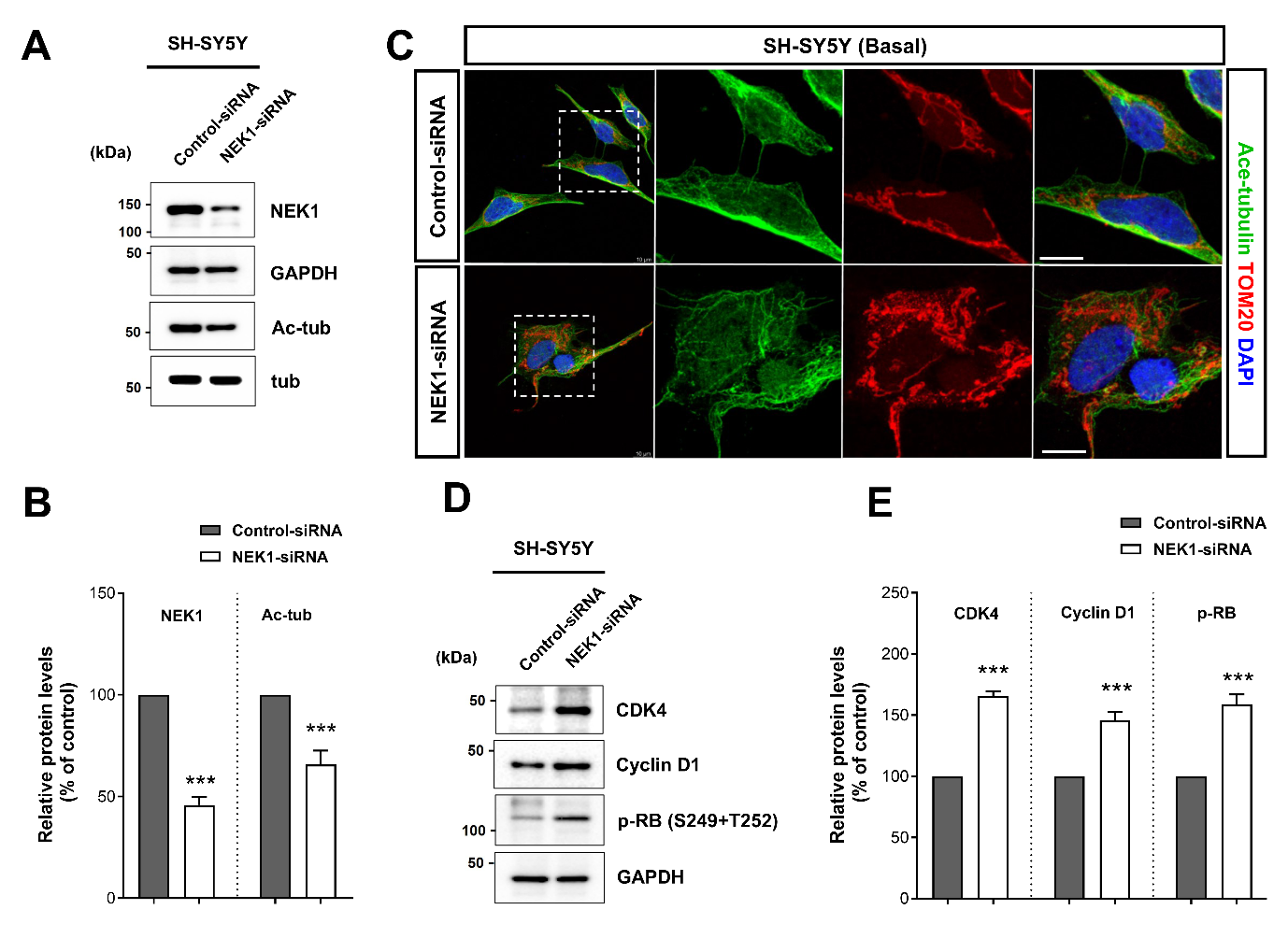


**Fig. S6.** **Knockdown (KD) of *NEK1* by siRNA in neuronal cells affects tubulin acetylation, mitochondrial distribution, and cell cycle progression.**

**A**. Western blot analysis of lysates from *NEK1* KD by siRNA in SH-SY5Y cells using anti-NEK1, anti-acetylated α-tubulin (Ac-tub), and anti-α-tubulin (tub) antibodies. GAPDH and tub were used as the loading controls. **B**. Quantitative analysis of densitometric measurements. NEK1 band intensities were normalized to GAPDH intensity. Band intensities of the Ac-tub were normalized to tub intensity. Data represent mean ± SEM (from three independent experiments). Comparisons were made against the control-siRNA (***P* < 0.01, ****P* < 0.001; Student’s *t-*test). **C**. Representative fluorescence images of primary cilia formation in SH-SY5Y cells by *NEK1* KD. Cells were stained with acetylated α-tubulin (green, ciliary axoneme marker), TOM20 (green, mitochondria marker), and DAPI (blue). The right panels show higher magnification views of the white box regions. Scale bar: 10 µm. **D**. Western blot analysis of serum-starved lysates from *NEK1* KD in SH-SY5Y cells using anti-CDK4, anti-Cyclin D1, and anti-p-pRB (S249+T252) antibodies. GAPDH was used as a loading control. **E**. Quantitative analysis of densitometric measurements. Band intensities were normalized to GAPDH intensity. Data represent mean ± SEM (from three independent experiments). Comparisons were made against the control-siRNA (****P* < 0.001; Student’s *t-*test).


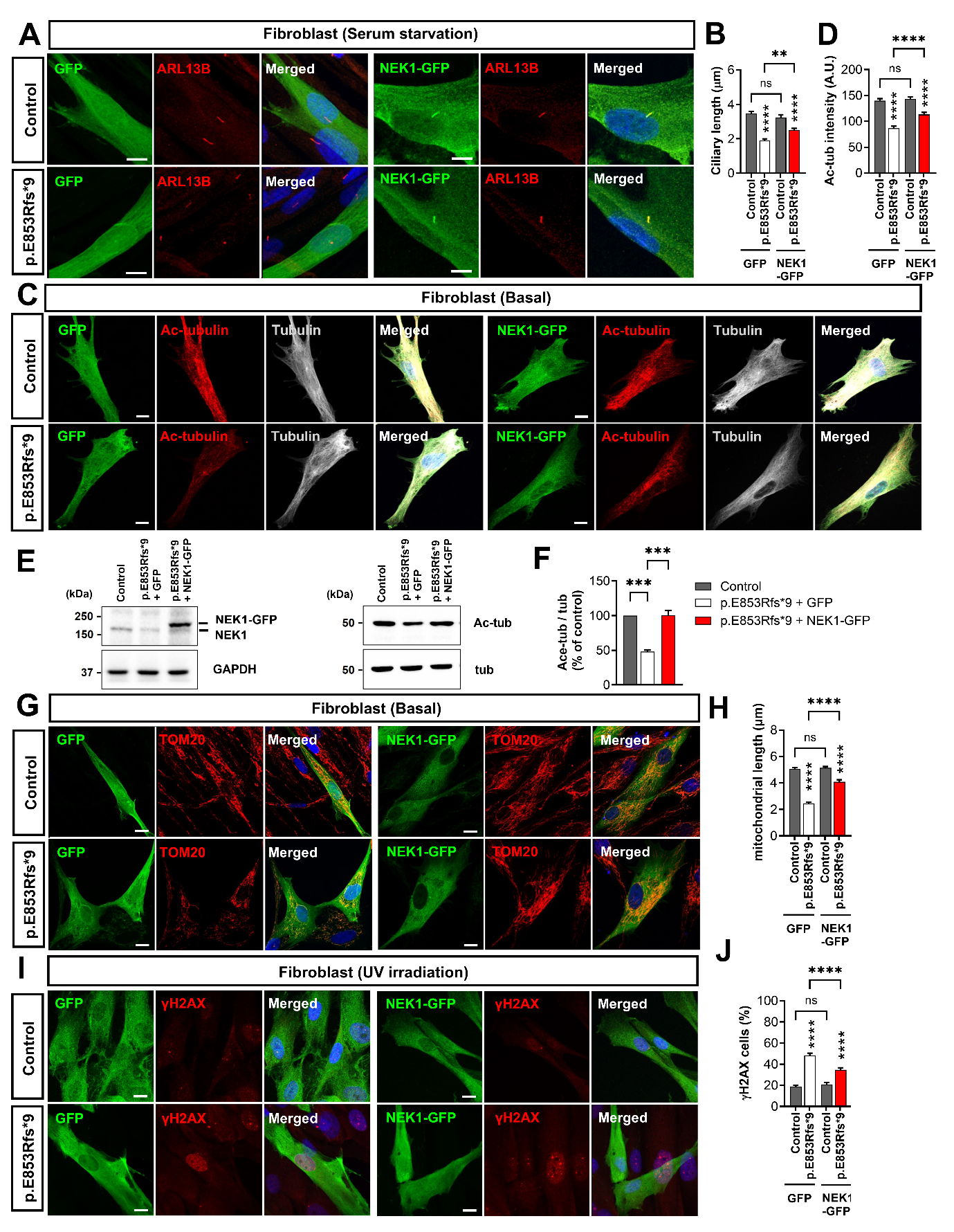


**Fig. S7.** **Overexpression of human NEK1 partially rescues the abnormalities in *NEK1*-LOF patient fibroblasts.**

**A.** Representative fluorescence images of primary cilia formation stained with anti-GFP (green), ARL13B (red, cilia marker), and DAPI (blue). Control and *NEK1*-LOF (p.E853Rfs*9) patient fibroblasts expressing NEK1-GFP or GFP were stimulated with serum starvation for 48 h. Scale bar: 10 µm. **B**. Quantification of the ciliary length in A. The 50 transfected cells per condition were quantified per replicate experiment (n = 3). Data represent mean ± SEM. Comparisons were made against the GFP control (ns: not significant, ***P* < 0.01, *****P* < 0.0001; one-way ANOVA with post-hoc Tukey’s tests). **C**. Representative fluorescence images of control and *NEK1*-LOF (p.E853Rfs*9) patient fibroblasts stained with anti-GFP (green), acetylated α-tubulin (Ac-tub, red), α-tubulin (gray), and DAPI (blue). Scale bar: 10 µm. **D**. Quantification of Ac-tub (red) intensity described in C. The 50 transfected cells per condition were quantified per replicate experiment (n = 3). Data represent mean ± SEM. Comparisons were made against the GFP control (ns, not significant, **P* < 0.05; one-way ANOVA with post-hoc Tukey’s test). **E**. Western blot analysis of lysates from control and *NEK1*-LOF (p.E853Rfs*9) patient fibroblasts expressing NEK1-GFP or GFP using anti-NEK1, anti-acetylated α-tubulin (Ac-tub), and anti-α-tubulin (tub) antibodies. GAPDH was used as the loading control. **F**. Quantification of acetylated α-tubulin (Ac-tub) normalized expression from three independent experiments. Ac-tub intensities were normalized to total α-tubulin (tub). Data represent mean ± SEM. Comparisons were made against the control (****P* < 0.001; one-way ANOVA with post-hoc Tukey’s tests). **G**. Representative fluorescence images of control and *NEK1*-LOF (p.E853Rfs*9) patient fibroblasts stained with anti-GFP (green), TOM20 (red, mitochondrial marker), and DAPI (blue). Scale bar: 10 µm. **H**. Quantification of the mitochondrial length in G. The 50 transfected cells per condition were quantified per replicate experiment (n = 3). Data represent mean ± SEM. Comparisons were made against the GFP control (ns: not significant, ***P* < 0.01, *****P* < 0.0001; one-way ANOVA with post-hoc Tukey’s tests). **I**. Representative fluorescence images of DNA damage response. Control and *NEK1*-LOF (p.E853Rfs*9) patient fibroblasts expressing NEK1-GFP or GFP were fixed 24 h after UV irradiation and stained with anti-GFP (green), γH2AX (red, DNA damage marker), and DAPI (blue). Scale bar: 10 µm. **J**. Quantification of the γH2AX-positive cells in I. The 50 transfected cells per condition were quantified per replicate experiment (n = 3). Data represent mean ± SEM. Comparisons were made against the GFP control (ns: not significant, **P* < 0.05, *****P* < 0.0001; one-way ANOVA with post-hoc Tukey’s tests).


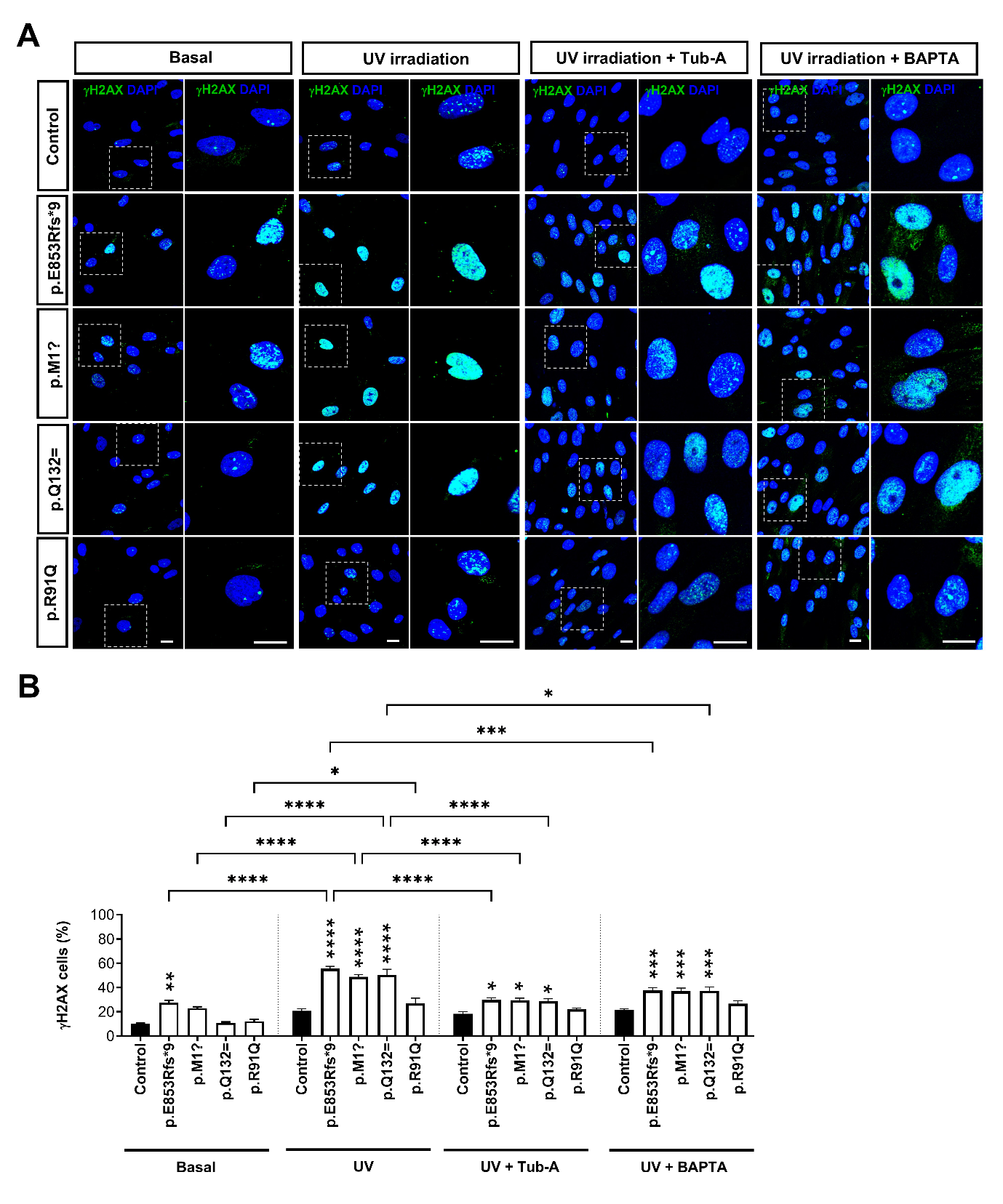


**Fig. S8.** **Impaired DNA damage repair in patient fibroblasts was partially rescued by treatment with tubastatin A or BAPTA.**

**A**. Representative fluorescence images of the DNA damage response in control and patient fibroblasts. Cells were pretreated with 1 μM tubastatin A (Tub-A) for 24 h or 10 µM of BAPTA for 60 min before UV irradiation and then stained with γH2AX (S139) (green, DNA damage marker) and DAPI (blue) either under basal condition (without UV irradiation) or after UV irradiation. The right panels illustrate higher magnification views of the white box regions. Scale bar: 10 µm. **B**. Quantification of the γH2AX-positive cells in each condition of A. The >100 cells per condition were quantified per replicate experiment (n = 3). Data represent mean ± SEM. Comparisons were made against the control (**P* < 0.05, ****P* < 0.001, *****P* < 0.0001; one-way ANOVA with post-hoc Tukey’s test).


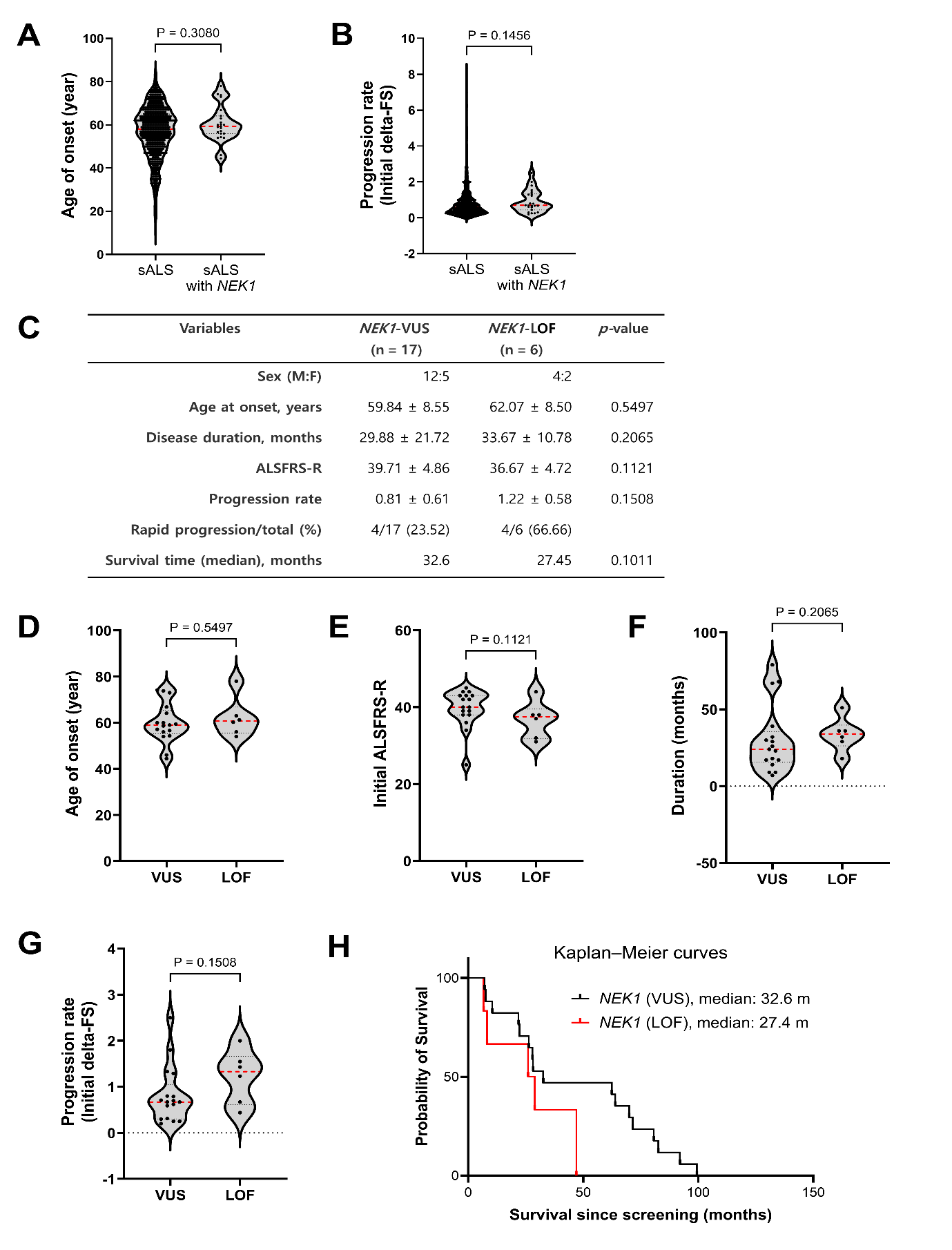


**Fig. S9. Comparmision of clinical characteristics of patients with *NEK1* variants.**

**A-B**. Comparison of clinical features with and without *NEK1* variants in patients with sALS; Age of onset (**A**), progression rate (**B**). **C-H**. Clinical features of sALS patients with *NEK1*-VUS variants and *NEK1*-LOF variants. Data are means (standard deviation).

Rapid progression was defined delta-FS ≥1.0. Survival time was calculated by considering the months from symptom onset to death or being tracheostomy-free (whichever occurred first) or the censoring date (the last follow-up day). Age of onset (**D**), initial ALSFRS-R score (**E**), duration (**F**), disease progression rate (**G**), Kaplan-Meier (KM) survival curve (**H**). The log-rank Mantel-Cox test was used for the KM survival curve, and a fixed date was used to censor data for survival analysis.

**Table S1**. Identified variants of the *NEK1* gene in ALS patients

| Patient ID | Nucleotide change^*^ | Amino acid  change | rs number | Allele frequency | | *In-silico*  Analysis | | | | | | ClinVar, | Classifi  -cation |
| --- | --- | --- | --- | --- | --- | --- | --- | --- | --- | --- | --- | --- | --- |
|  |  |  |  | gnomAD (total)^†^ | gnomAD (EAS)^†^ | SIFT | Poly Phen-2 | Meta SVM | Meta LR | REVEL | SpliceAI  (delta score) |  |  |
| 1730 | c.1A>G | p.M1? | N/A | < 0.00001 | N/A | D | PD | T | T | 0.642 | N/A | - | VUS |
| 1836 | c.29T>C | p.I10T | rs753341812 | 0.00001 | 0 | D | PD | D | T | 0.739 | N/A | - | VUS |
| 2226, 1898 | c.259G>T | p.D87Y | N/A | N/A | N/A | D | PD | T | T | 0.632 | N/A | - | VUS |
| 1454 | c.272G>A | p.R91Q | rs750846188 | 0.00001 | 0.00002 | D | PSD | T | T | 0.279 | N/A | - | VUS |
| 2124, 1628 | c.396G>A | p.Q132= | N/A | N/A | N/A | N/A | N/A | N/A | N/A | N/A | 0.36 | - | VUS |
| 2066, 2505, 2374 | c.445A>C | p.I149L | rs1372039005 | < 0.00001 | 0.00020 | D | PD | T | T | 0.345 | N/A | - | VUS |
| 2245 | c.464+8G>T | N/A | rs767640659 | < 0.00001 | 0.00007 | N/A | N/A | N/A | N/A | N/A | 0 | - | VUS |
| 1757 | c.899T>C | p.I300T | rs753392280 | 0.00002 | 0.00047 | T | B | T | T | 0.397 | N/A | - | VUS |
| 2601 | c.908T>C | p.M303T | rs756685207 | < 0.00001 | 0 | T | B | T | T | 0.059 | N/A | - | VUS |
| 2090 | c.1823G>A | p.R608H | rs551275723 | 0.00002 | 0.00049 | D | PD | D | D | 0.732 | N/A | VUS/LB | VUS |
| 1428 | c.1928G>A | p.R643H | rs776789588 | 0.00004 | 0.00025 | D | B | T | D | 0.634 | N/A | VUS | VUS |
| 2523 | c.2352T>A | p.D784E | rs368611367 | N/A | 0 | T | B | T | T | 0.552 | N/A | - | VUS |
| 1696, 1988, 2419, 2782 | c.2555dup | p.E853Rfs*9 | rs757133826 | < 0.00001 | 0.00007 | N/A | N/A | N/A | N/A | N/A | N/A | - | VUS |
| 2230 | c.3051G>T | p.K1017N | N/A | N/A | N/A | D | PSD | T | T | 0.574 | N/A | - | VUS |
| 1955 | c.3222+1G>A | N/A | rs778672440 | < 0.00001 | 0.00002 | N/A | N/A | N/A | N/A | N/A | 0.83 | - | VUS |
| 2909 | c.3336del | p.D1112Efs*50 | N/A | N/A | N/A | N/A | N/A | N/A | N/A | N/A | N/A | - | LPV |

Abbreviations: N/A, not applicable; B, benign; D, deleterious; EAS, East Asian; LPV, likely pathogenic variant; PD, probably damaging; PSD, possibly damaging; VUS, variant of uncertain significance; LB, likely benign

*Nucleotides are numbered according to the reference cDNA sequence, GenBank accession number NM_001199397.3

^†^gnomAD, gnome Aggregation Database (<http://gnomad.broadinstitute.org/>, ver.4.1.1)

^‡^ClinVar (<https://www.ncbi.nlm.nih.gov/clinvar>, accessed on 05/02/2025)

**Table S2**. Demographic and clinical characteristics of patients with *NEK1* variants

| MND | cDNA | Amino acid change | Sex | Age (y) | Age of onset (y) | Initial ALSFRS-R* | Duration from onset to latest visit (m) | Initial delta FS† | Site of onset | Survival time (m) ¶ | Outcome | Family history ‡ | Dementia | Cognitive or behavioral impairment |
| --- | --- | --- | --- | --- | --- | --- | --- | --- | --- | --- | --- | --- | --- | --- |
| 1428 | c.1928G>A | p.Arg643His | M | 60.8 | 59.4 | 38 | 23 | 0.59 | L | 22.3 | D | No | No | No |
| 1454 | c.272G>A | p.Arg91Gln | M | 68.5 | 66.8 | 34 | 67 | 0.7 | L | 71.5 | D | No | No | Bilateral frontal and memory dysfunction, severe depression |
| 1628 | c.396G>A | p.Gln132= | M | 57.7 | 57.1 | 44 | 39 | 0.67 | U | 82.6 | D | No | No | Bilateral frontal dysfunction |
| 1696 | c.2555dup | p.Glu853Argfs*9 | M | 55.1 | 54 | 32 | 29 | 1.23 | L | 26.0 | D | No | No | Bilateral frontal and left parietal dysfunction |
| 1730 | c.1A>G | p.Met1? | M | 60.6 | 61.2 | 38 | 32 | 1.43 | U | 8.2 | D | No | No | Left frontal dysfunction |
| 1757 | c.899T>C | p.Ile300Thr | M | 48.0 | 46.0 | 42 | 32 | 0.3 | L | 62.4 | D | No | No | No |
| 1836 | c.29T>C | p.Ile10Thr | M | 74.5 | 74.2 | 44 | 17 | 0.8 | B | 7.1 | D | No | No | No |
| 1898 | c.259G>T | p.Asp87Tyr | F | 75.0 | 73.8 | 43 | 24 | 0.31 | B | 28.2 | D | No | No | No |
| 1955 | c.3222+1G>A | - | F | 60 | 59 | 40 | 14 | 1.33 | L | 7.6 | D | No | No | Bilateral frontal and left temporal lobe dysfunction |
| 1988 | c.2555dup | p.Glu853Argfs*9 | M | 57 | 56 | 38 | 36 | 2 | U/L | 6.7 | D | No | No | No |
| 2066 | c.445A>C | p.Ile149Leu | M | 55.8 | 54.3 | 36 | 26 | 0.67 | B | 26.3 | D | No | No | No |
| 2090 | c.1739G>A | p.Arg580His | M | 45.6 | 44.4 | 40 | 79 | 0.62 | L | 99.4 | A | No | No | No |
| 2124 | c.396G>A | p.Gln132= | M | 64.3 | 64.1 | 43 | 9 | 2.5 | B | 10.5 | D | No | No | Bilateral frontal dysfunction |
| 2226 | c.259G>T | p.Asp87Tyr | F | 62 | 60 | 38 | 17 | 0.71 | U | 21.9 | D | No | Yes | bvFTD |
| 2245 | c.464+8G>T | N/A | M | 57.4 | 56.6 | 39 | 9 | 1.29 | U | 92.0 | D | No | No | No |
| 2230 | c.3051G>T | p.Lys1017Asn | F | 59 | 56 | 43 | 68 | 0.2 | U | 32.6 | D | No | No | No |
| 2374 | c.445A>C | p.Ile149Leu | M | 75.0 | 73.0 | 25 | 29 | 0.79 | R | 27.9 | D | No | Yes | Alzheimer dementia |
| 2419 | c.2555dup | p.Glu853Argfs*9 | M | 62.0 | 60.2 | 37 | 51 | 0.44 | L | 47.0 | A | No | No | No |
| 2505 | c.445A>C | p.Ile149Leu | M | 61 | 60 | 45 | 18 | 0.25 | U | 80.7 | A | No | No | Left frontal dysfunction |
| 2523 | c.2352T>A | p.Asp784Glu | M | 60.6 | 58.6 | 42 | 30 | 0.25 | L | 63.9 | D | No | No | Bilateral frontal dysfunction |
| 2601 | c.908T>C | p.Met303Thr | F | 55 | 54 | 39 | 7 | 1.8 | U | 70.0 | A | No | No | No |
| 2782 | c.2555dup | p.Glu853Argfs*9 | F | 63 | 63 | 44 | 36 | 0.67 | U | 47.0 | D | No | No | Left frontal dysfunction |
| 2909 | c.3336del^§^ | p.Asp1112Glufs*50 | F | 80 | 78 | 31 | 18 | 1.55 | B | 28.9 | D | No | No | Bilateral frontal and left parietal lobe dysfunction |

Abbreviations: ALSFRS-R, Amyotrophic Lateral Sclerosis Functional Rating Scale-Revised (an indicator of the disease progression in patients with ALS); N/A, not applicable; bvFTD, behavioral variant frontotemporal dementia; y, years; m, months; A, alive; D, died; U, upper limbs; L, lower limbs; B, bulbar; R, respiratory, F, female; M, male

*ALSFRS-R scores evaluated at the time of DNA sampling.

† The disease progression rate (48 – ALSFRS-R at the time of initial visit]/duration from onset to initial visit).

‡ Family history (ALS or FTD)

§ Comorbid Mutations; CHRNA4 c.1310C>T, p.Ala437Val, TAF15 c.1320_1346dup, p.Ser451_Arg459dup

¶ Survival time was calculated by considering the months from symptom onset to death or being tracheostomy-free (whichever occurred first) or the censoring date (the last follow-up day; 05/02/2025).
